# Comprehensive genomic profile of Chinese lung cancer patients and mutation characteristics of individuals resistant to icotinib/gefitinib

**DOI:** 10.1038/s41598-020-76791-y

**Published:** 2020-11-20

**Authors:** Yanhong Shang, Xiaofang Li, Weiwei Liu, Xiaoliang Shi, Shaohua Yuan, Ran Huo, Guotao Fang, Xiao Han, Jingnan Zhang, Kunjie Wang, Zhengyue Dou, Yan Zhang, Aimin Zang, Lin Zhang

**Affiliations:** 1 Department of Medical Oncology, Affiliated Hospital of Hebei University, No. 648, Dongfeng East Road, Baoding City, 071000 Hebei People’s Republic of China; 2 OrigiMed Co. Ltd, Shanghai, 201114 People’s Republic of China; 3 Department of Oncology, The First Hospital of Shijiazhuang City, No.36, Fanxi Road, Chang’an District, Shijiazhuang City, 050000 Hebei People’s Republic of China

**Keywords:** Cancer, Biomarkers, Oncology

## Abstract

Lung cancer is the leading causes of cancer-related death worldwide. Precise treatment based on next-generation sequencing technology has shown advantages in the diagnosis and treatment of lung cancer. This cohort study included 371 lung cancer patients. The lung cancer subtype was related to the smoking status and sex of the patients. The most common mutated genes were *TP53* (62%), *EGFR* (55%), and *KRAS* (11%). The mutation frequencies of *EGFR*, *TP53*, *PIK3CA*, *NFE2L2*, *KMT2D*, *FGFR1*, *CCND1*, and *CDKN2A* were significantly different between lung adenocarcinoma and lung squamous cell carcinoma. We identified the age-associated mutations in *ALK*, *ERBB2*, *KMT2D*, *RBM10*, *NRAS*, *NF1*, *PIK3CA*, *MET*, *PBRM1*, *LRP2*, and *CDKN2B*; smoking-associated mutations in *CDKN2A*, *FAT1*, *FGFR1*, *NFE2L2*, *CCNE1*, *CCND1*, *SMARCA4*, *KEAP1*, *KMT2C*, and *STK11*; tumor stage-associated mutations in *ARFRP1*, *AURKA*, and *CBFB*; and sex-associated mutations in *EGFR*. Tumor mutational burden (TMB) is associated with tumor subtype, age, sex, and smoking status. TMB-associated mutations included *CDKN2A*, *LRP1B*, *LRP2*, *TP53*, and *EGFR*. *EGFR* amplification was commonly detected in patients with acquired lcotinib/gefitinib resistance. *DNMT3A* and *NOTCH4* mutations may be associated with the benefit of icotinib/gefitinib treatment.

## Introduction

Lung cancer, the most common type of cancer, has the largest number of new cases and deaths worldwide^1^. Non-small cell lung cancer (NSCLC), including lung adenocarcinoma (LUAD) and lung squamous cell carcinoma (LUSC), is the most common subtype of lung cancer^2^. The conventional treatment for lung cancer involves surgical resection, radiotherapy, and chemotherapy. Unfortunately, most patients are diagnosed at an advanced stage of the disease and miss the best treatment time, which leads to poor prognosis^3^. Early diagnosis and precise treatment are still the main obstacles in lung cancer treatment.

The continuous development of sequencing technology enables large-scale tumor- related gene detection. The mutational landscape of lung cancers, including LUSC and LUAD, has been reported by The Cancer Genome Atlas (TCGA)^4,5^. Molecular targeted therapy and immunotherapy have the advantages of high accuracy, high conformability and fewer side effects^6,7^. Ding et al. also compared the mutational features of LUSC and LUAD in Chinese NSCLC patients^8^. The understanding of molecular mutation characteristics in a large proportion of lung cancer patients may allow the development of personalized molecular targeted therapy or immunotherapy for patients with target mutations^9^.

Several genomic alterations that could be relevant in the clinical management of patients, such as *RET*, *ALK*, and *NTRK1* fusions, and *EGFR* and *KRAS* mutations, were identified and used for the exploration of targeted drugs^10–17^. At present, targeted treatment of *EGFR* mutant tumors with EGFR tyrosine kinase inhibitors (TKIs) has been used as the standard clinical treatment^18–20^. However, cancer cells often develop resistance to these drugs, which may lead to progression^21^. Acquired EGFR T790M mutation is one reason for resistance to first- and second-generation EGFR-TKIs^22^. Osimertinib, a targeted drug to treat NSCLC patients with certain mutations demonstrated improved efficacy for EGFR T790M mutation^23^. However, existing targets and targeted drugs are far from the cure for all lung cancer.

The different mutational characteristics of lung cancer patients from different regions affects the efficacy of target drugs. Satio et al. reported differences in driver gene aberration frequencies between Japanese and American patients^6^. This indicats different guiding effects of targeted drugs on the American and Asian populations. At present, most studies are focused on populations in Europe, America, Japan and South Korea^10,13,15,16,24^. The study of the mutational profile of lung cancer patients in China may identify the genetic heterogeneity that is of great significance to the study of targeted therapy for Chinese patients.

In this study, we acquired 371 lung cancer tissue samples from Chinese patients at the Affiliated Hospital of Hebei University and determined their comprehensive genomic profiles to provide evidence for the potential development of special therapeutic targets and to identify new prognostic biomarkers.

## Materials and methods

### Ethics statement

This project was approved by the Ethics Committees of the Affiliated Hospital of Hebei University (approval number: HDFY-LL-2019-064). We declare that all methods used in this protocol were carried out in accordance with relevant guidelines and regulations. All patients and all participants provided informed consent.

### Patients and sample collection

A total of 371 Chinese lung cancer patients were enrolled in this study from the Affiliated Hospital of Hebei University. Formalin-fixed paraffin-embedded (FFPE) tumor tissues and matched blood samples were collected and transferred to the laboratory of OrigiMed (Shanghai, China), which certified by College of American Pathologists and Clinical Laboratory Improvement Amendments, for genetic variation detection. Referring to previous methods^25,26^, the genomic DNA was prepared using the QIAamp DNA FFPE Tissue Kit and QIAamp DNA Blood Midi Kit (Qiagen, Hilden, Germany) according to the manufacturer’s instructions. The concentration of DNA was measured by Qubit and normalized to 20–50 ng/μL for sequencing.

### Identification of genomic alterations and tumor mutational burden

Genomic mutations were identified using the next-generation sequencing (NGS)-based YuanSu450 gene panel (OrigiMed, Shanghai, China), which covers all coding exons of 450 cancer-related genes that are frequently rearranged in solid tumors. The genes were captured and sequenced with a mean depth of 800 × using Illumina NextSeq 500 (Illumina, Inc., San Diego, CA, USA). The procedures followed the steps described by Frampton et al.^27^ Genomic alterations (GAs) were identified according to previous study^28^.Single nucleotide variants (SNVs) were identified using MuTect (version 1.7, Broad Institute, Cambridge, MA, USA). Insertion-deletions (Indels) were identified using PINDEL (version 0.2.5). The functional impact of GAs was annotated using SnpEff3.0. Copy number variation regions were identified using Control-FREEC (version 9.7, Institute Cochin, Paris, France) with the following parameters: window = 50,000 and step = 10,000. Gene fusions were detected using an in-house developed pipeline. Gene rearrangements were assessed using Integrative Genomics Viewer. Tumor mutational burden (TMB) was estimated by counting the coding somatic mutations, including SNVs and Indels, per megabase of the sequence examined in each patient. The TMB value was further divided into two groups: TMB-H, defined as ≥ 10 mutations/Mb; TMB-L, defined as < 10 mutations/Mb.

### Statistical analysis

Statistical analyses were performed using SPSS version 22.0 (SPSS Inc., Chicago, IL, USA). Fisher’s exact test was used for the association analysis of categorical variables. The associations of normally and non-normally distributed data were analyzed using Student's *t*‑test and Wilcoxon rank test respectively. The Kruskal–Wallis test was used to analyze the association between multiple groups of non-parametric data. P < 0.05 was considered statistically significant.

## Results

### Clinical characteristics of LC patients

The 371 Chinese lung cancer patients included 187 males and 184 females with a median age of 62 years (range, 27–84 years). The smoking status of 180 patients were collected, including 70 (18.87%) patients with a history of smoking > 10 years, and 110 (29.65%) who had never smoked. Among the smokers, 67 (95.7%) were male and 3 (4.3%) were female. Among non-smokers, 27 (24.5%) were male and 83 (75.5%) were female.

According to pathological classification, there were 335 (90.30%) NSCLCs, 11 (2.97%) small cell lung cancers (SCLCs), and 25 (6.74%) unclassified lung cancers in this cohort. The NSCLC consisted of 286 LUADs, 30 LUSCs, 8 LASCs, and 11 unclassified NSCLC (Table 1). Patients tumors were divided into stages I-IV based on the 8th edition Classification for lung cancer^29^ and consisted of 56 stage I, 24 stage II, 50 stage III, 180 stage IV, and 61 unclear tumor stage samples (Table 1). Of all 371 samples used for NGS testing, 297 were from primary lesions, 56 were from metastases lesions—26 pleural (7.0%), 9 lymphatic (2.43%), 9 bone (2.43%), 3 liver (0.81%), 2 brain (0.54%), 2 peritoneal (0.54%), 1 adrenal gland (0.27%), and 4 unclear (1.08%)—and 18 samples were of unknown origin (Table 1).

**Table 1:** Clinicopathologic features of this cohort.

Total		371
Gender (n/%)	Male	187 (50.40%)
	Female	184 (49.60%)
Age	Median (range)	62 (27–84)
Smoking status (n/%)	Smoking	70 (18.87%)
	Nonsmoking	110 (29.65%)
	Unknown	191 (51.48%)
TMB	Median (range)	3 (0–55.7)
Tumor sites (n/%)	Primary lesion	297 (80.05%)
	Metastases lesion	56 (15.09%)
	Pleural	26 (7.0%)
	Lymphatic	9 (2.43%)
	Bone	9 (2.43%)
	Liver	3 (0.81%)
	brain	2 (0.54%)
	peritoneal	2 (0.54%)
	Adrenal gland	1 (0.27%)
	Unclear	4 (1.08%)
	Unknown	18 (4.85%)
Tumor stage (n/%)	I	42 (11.32%)
	II	30 (8.09%)
	III	46 (12.40%)
	IV	121 (32.61%)
	Unknown	132 (35.58%)
Tumor subtype (n/%)	Non-small cell lung cancer	335 (90.30%)
	Lung adenocarcinoma	286
	Lung squamous cell carcinoma	30
	Lung adenosquamous carcinoma	8
	Unknown	11
	Small cell lung cancer	11 (2.97%)
	Unknown	25 (6.74%)

*TMB* tumor mutational burden.

### Correlations between tumor subtype and smoking status, sex, tumor stage, and age of patients

**Figure 1:**
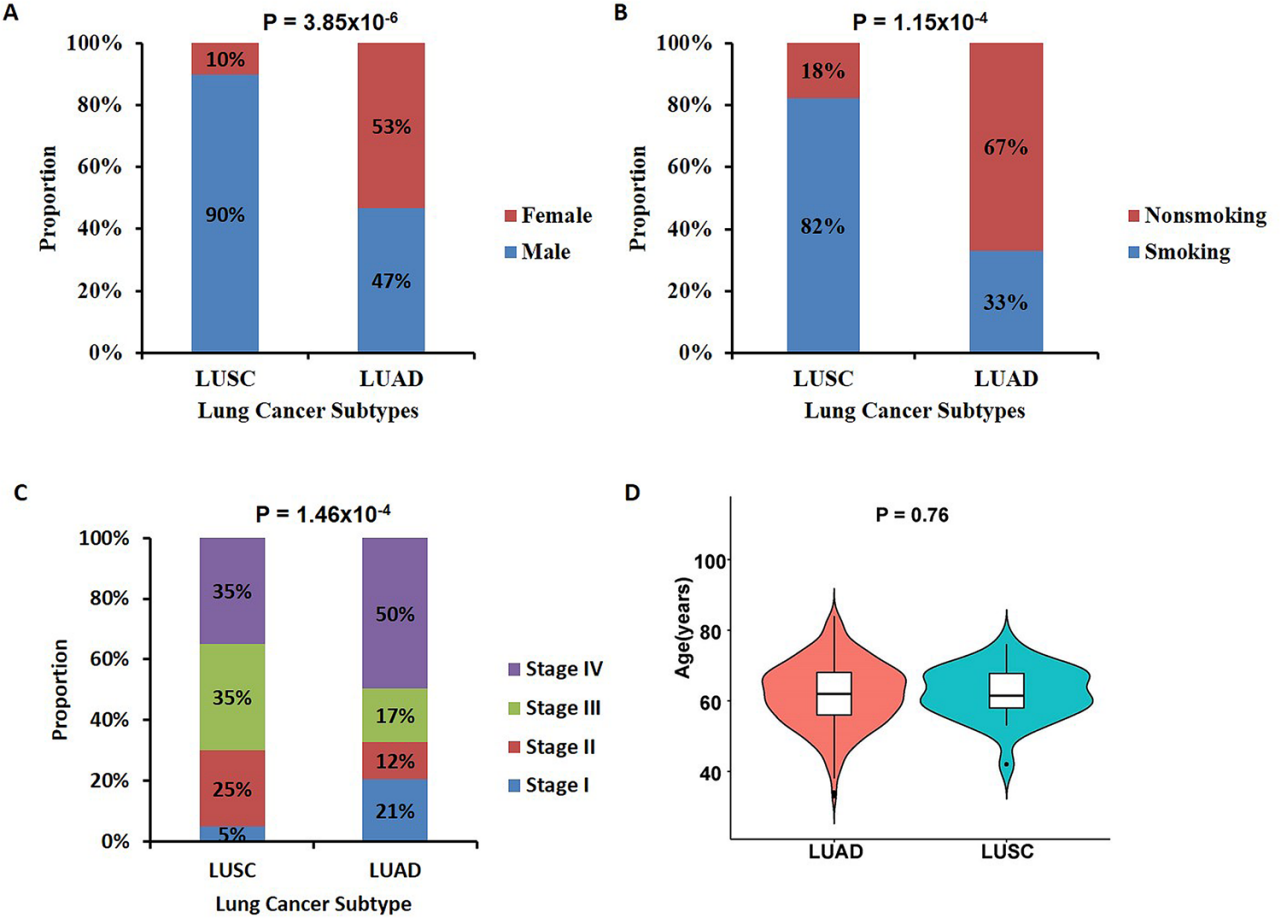
Association between tumor subtypes and clinical characteristics. (**A**) Association between tumor subtypes and sex; (**B**) Association between tumor subtypes and smoking status; (**C**) Correlation between tumor subtypes and tumor stage; (**D**) Correlation analysis between tumor subtypes and patient age. *LUAD* lung adenocarcinoma, *LUSC* lung squamous cell carcinoma, *LASC* lung adenosquamous cell carcinoma, *SCLC* small cell lung cancer.

Based on clinical information, we identified the correlation between tumor type and sex, smoking history, age, and tumor stage. Considering the small number of SCLC and LASC cases, we excluded them from the correlation analysis. The proportion of patients with a smoking history was higher than those who never smoked in LUSC, while the proportion of patients who never smoked was higher than those with a smoking history in LUAD. Statistical analysis showed that smoking history was correlated with tumor subtype (Fig. 1A). In addition, we found that the proportion of male patients was higher than that of female patients with LUSC, while the proportion of female patients was higher than that of male patients with LUAD. Statistical analysis showed that the sex of patients was correlated with tumor subtype (Fig. 1B). Meanwhile, we found that the proportion of stage IV tumors was high in LUAD, while the proportion of stage I tumors was high in LUSC. Statistical analysis showed a significant association between tumor stage and tumor subtype (Fig. 1C). In addition, we found that the majority of patients were near 60 years old cross different tumor subtypes. Our results showed that there was no correlation between tumor subtype and patient age (Fig. 1D).

### Genomic alterations in this cohort

**Figure 2:**
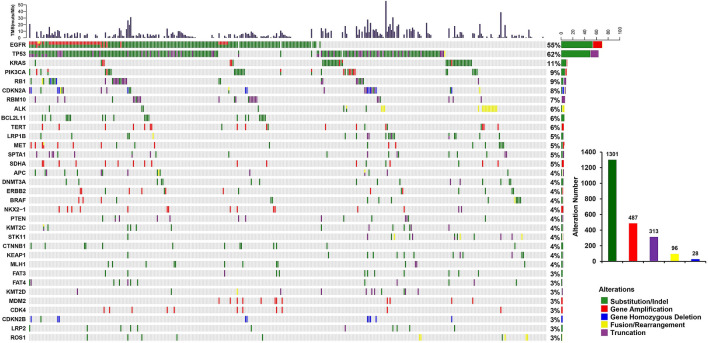
Mutational landscape of 371 Chinese lung cancer patients. The X-axis represents each patient tissue sample and the Y-axis represents each mutated gene. The bar graph above shows the tumor mutational burden (TMB) value of each sample, and the bar graph on the right shows the mutation frequency of each mutated gene. Statistical distribution of variation types is shown in the right column. Green represents substitution/indel, red represents gene amplification, blue represents gene homozygous deletion, yellow represents fusion/rearrangement, and purple represents truncation mutations.

A total of 2225 clinically relevant GAs were identified in this cohort, with a mean of 6.0 alterations per sample in 387 genes. Among these mutations, 1301 (68.5%) were substitution/indels, 487 (21.9%) were gene amplifications, 313 (14.1%) were truncations, 96 (4.3%) were fusions/rearrangements, and 28 (1.3%) were gene homozygous deletions (Fig. 2, Table S1). The most common mutated genes were *EGFR* (55%, 203/371), *TP53* (62%, 228/371), and *KRAS* (11%, 41/371). The most common gene fusion/rearrangement was *ALK*, and the main amplifications included *EGFR*, *PIK3CA*, *TERT*, *MET*, *SDHA*, *ERBB2*, *BRAF*, *NKX2*-1, *MDM2* and *CDK4* (Fig. 2, Table S1). In the same patient, one mutation site was detected in most genes, while two or three mutations were detected in a few genes such as *EGFR*, *TP53*, *KRAS*, *PIK3CA*, *ALK*, and *MET*.

In 203 lung cancer tissue samples, we detected a total of 313 EGFR GAs: 253 Substitution/Indels (including 250 Substitutions/Shortindels and 3 LongIndels, with 5 SNVs were belonging to germline mutation), 58 gene amplifications, and 2 fusions. The most common alteration was L858R, and followed by exon 19 deletion (19del) (Fig. S1).

**Figure 3:**
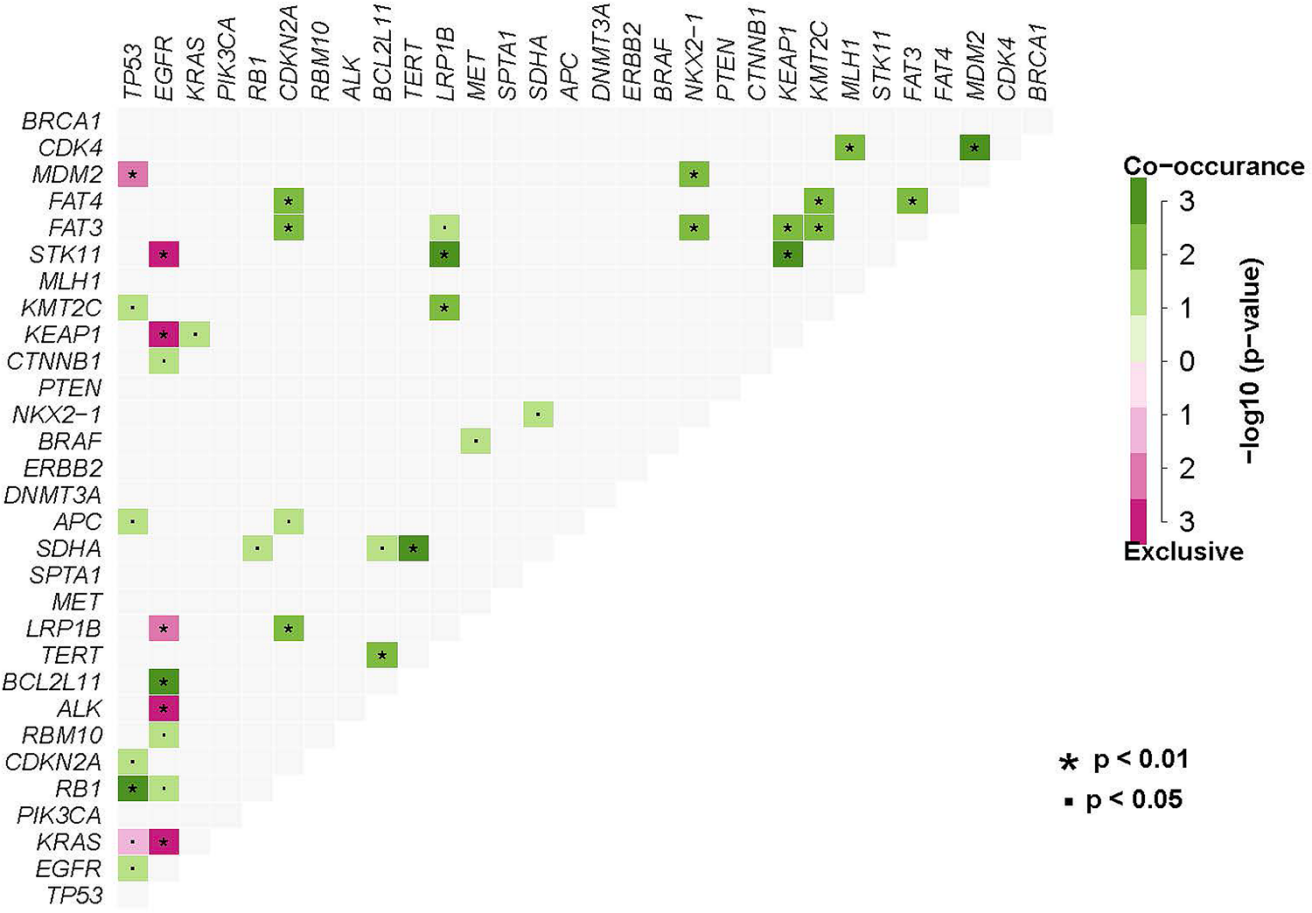
Co-occurring analysis of Chinese lung cancer patients. Green represents the co-occurrence mutations; pink represents the mutual exclusive mutations. ^▪^P < 0.05, *P < 0.01.

The co-occurrence and mutual exclusion of gene mutations can influence prognosis. For this reason, we performed a co-mutation analysis of this cohort. Our results showed that mutations in *KMT2C*, *APC*, *CDKN2A*, *RB1*, and *EGFR* co-occurred with *TP53* mutations, while mutations in *MDM2* and *KRAS* were mutually exclusive with *TP53* mutations. In addition, the mutations in *BCL2L11*, *CTNNB1*, *RBM10*, and *RB1* co-occurred with *EGFR* mutations, while mutations in *STK11*, *KEAP*, *LRP1B*, *ALK*, and *KRAS* were mutually exclusive with *EGFR* mutations. Notably, both *TP53* and *EGFR* mutations co-occurred with *RB1* mutations, and mutually exclusive with *KRAS* mutations (Fig. 3).

### Differences between lung adenocarcinoma and lung squamous cell carcinoma

**Figure 4:**
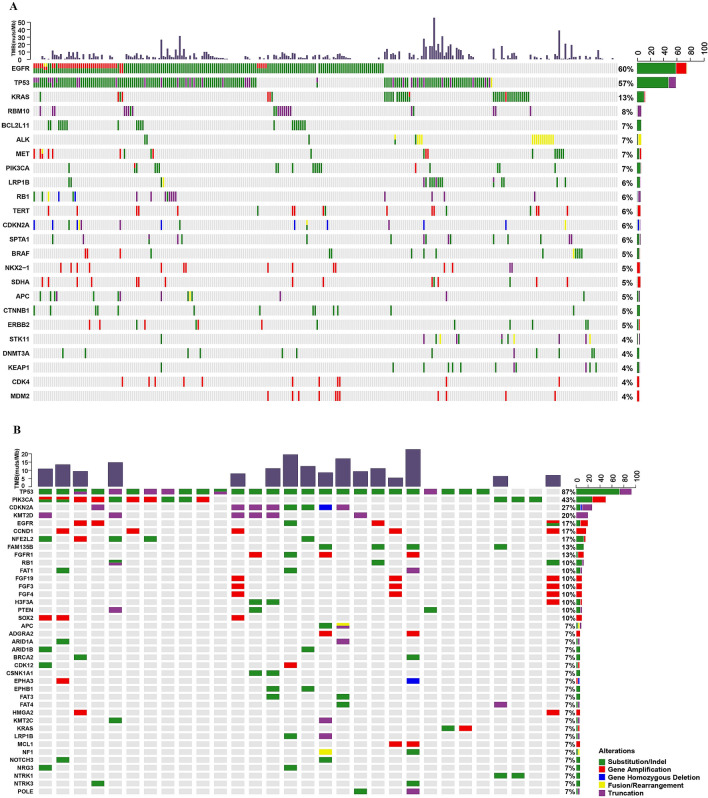
Mutational landscape of Chinese patients with lung adenocarcinoma (**A**) and lung squamous cell carcinoma (**B**). The X-axis represents each patient tissue sample and the Y-axis represents each mutated gene. The bar graph above shows the tumor mutational burden (TMB) value of each sample, and the bar graph on the right shows the mutation frequency of each mutated gene. Green represents substitution/Indel, red represents gene amplification, blue represents gene homozygous deletion, yellow represents fusion/rearrangement, and purple represents truncation mutations.

**Figure 5:**
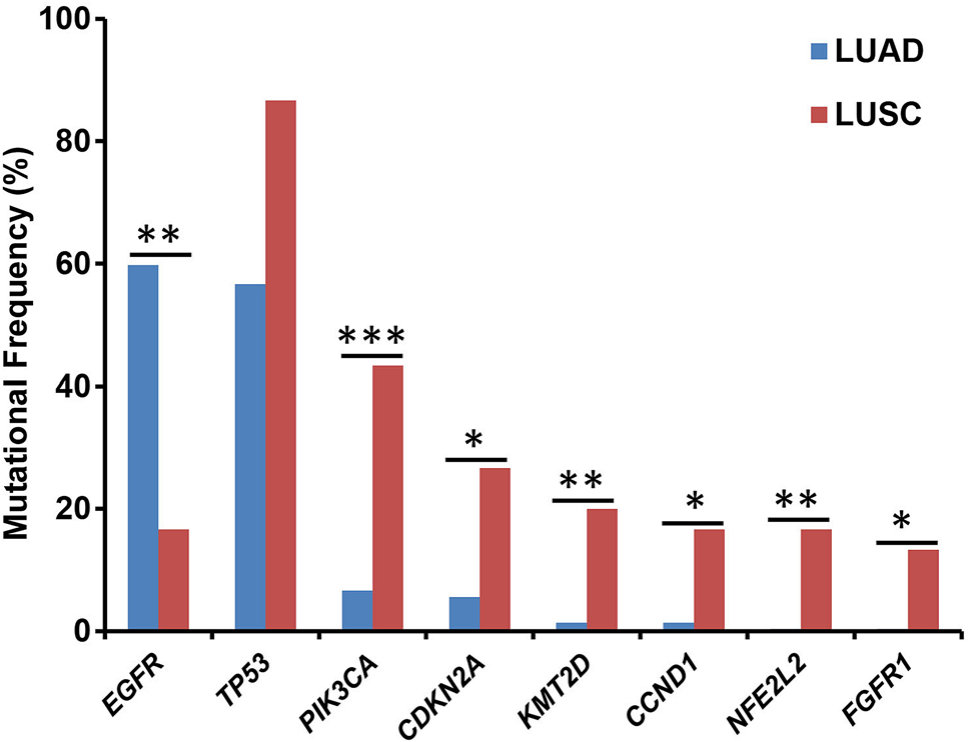
Correlation analysis of mutated genes and tumor subtype. The X-axis shows the mutated genes and the Y-axis represents the mutational frequency of each gene. LUAD and LUSC are represented by blue and red, respectively. *P < 0.05, **P < 0.01, and ***P < 0.001. *LUAD* lung adenocarcinoma, *LUSC* lung squamous cell carcinoma.

In this study, NSCLC represented nearly 90% of cases and mainly consisted of LUAD and LUSC. As shown in Fig. 4, there were many differences in the molecular characteristics of LUAD and LUSC. The most common mutations in LUAD and LUSC were *EGFR*, *TP53*, and *KRAS,* and *TP53*, *PIK3CA*, *CDKN2A*, *EGFR*, *CCND1*, *NFE2L2*, *FAM1358*, and *FGFR1*, respectively (Fig. 4A,B). In LUAD, the main mutation type of *PIK3CA* was SNV; in LUSC, it was mainly gene amplification. *ALK* fusion; *RBM10* truncation; *MET*, *TERT*, *NKX2-1*, *SDHA*, *CDK4*, and *MDM2* amplifications, and *CDKN2A* deletion were mainly identified in LUAD (Fig. 4A). *CCND1*, *FGFR1*, and *FGF3*/*4/13*, and *SOX2* amplifications were mainly identified in LUSC (Fig. 4B). Statistical analysis showed that the mutation frequency of *EGFR* was higher in LUAD than in LUSC (*P* = 0.0015), while the mutation frequencies of *TP53* (*P* = 0.064), *PIK3CA* (*P* = 0.00014), *NFE2L2* (*P* = 0.0038), *KMT2D* (*P* = 0.0066), *FGFR1* (*P* = 0.023), *CCND1* (*P* = 0.033), and *CDKN2A* (*P* = 0.035) were higher in LUSC than in LUAD (Fig. 5).

### Age-related gene mutations in Chinese lung cancer patient

**Figure 6:**
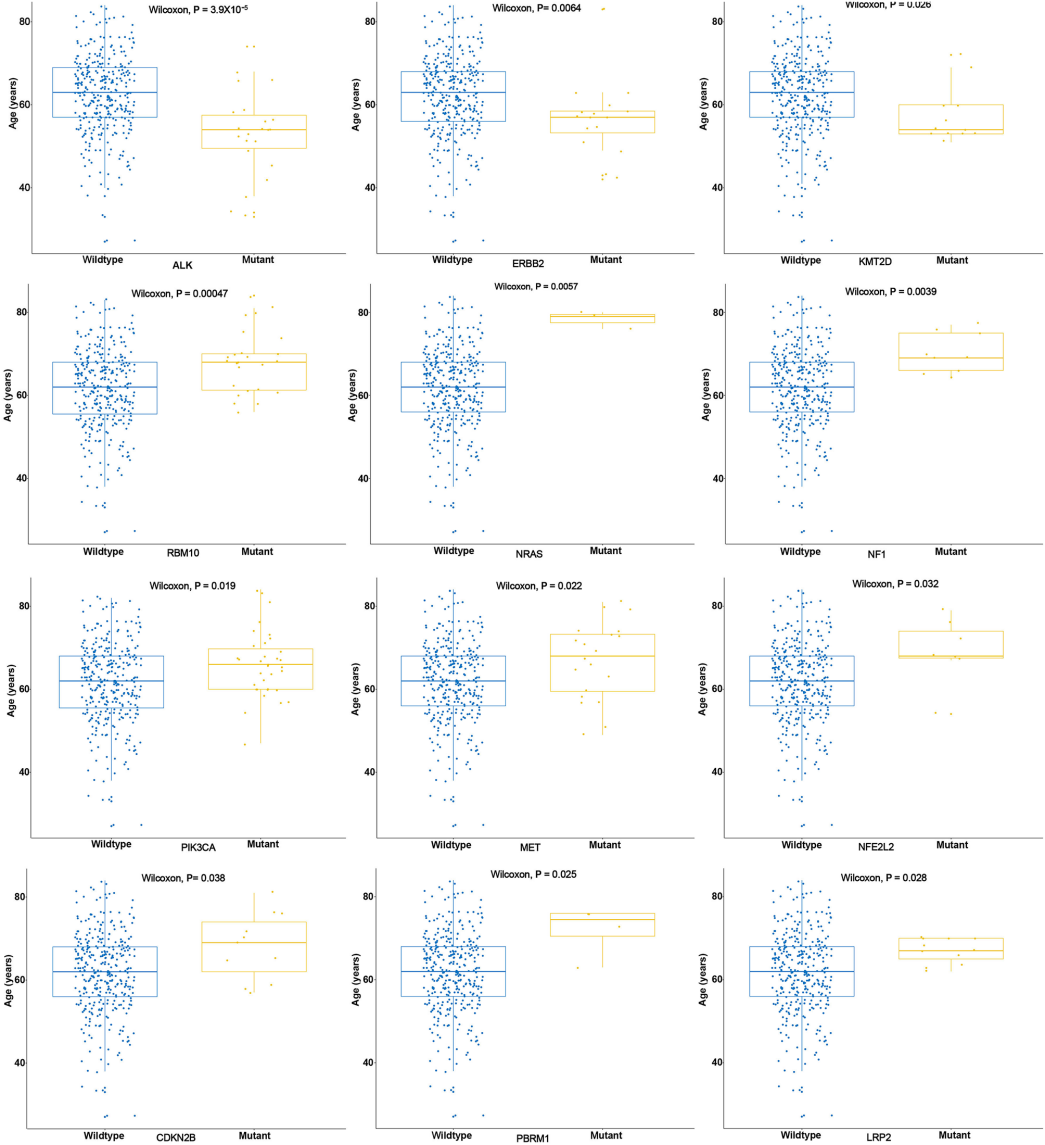
Correlation analysis of mutated genes and patients’ age. The X-axis shows the mutated genes and the Y-axis represent the patients’ age.

We examined the correlation between patient age and gene mutations. The results showed that patients with mutations in *ALK*, *ERBB2*, or *KMT2D* were younger than those without these mutations, while patients with mutations in *RBM10*, *NRAS*, *NF1*, *PIK3CA*, *MET*, *PBRM1*, *LRP2*, *NFE2L2*, or *CDKN2B* were older than those without these mutations. Statistical analysis showed that the mutation of these genes was significantly associated with the patient age (Fig. 6).

### Correlations between mutated genes and smoking status, tumor stage, and sex in Chinese lung cancer patients

**Figure 7:**
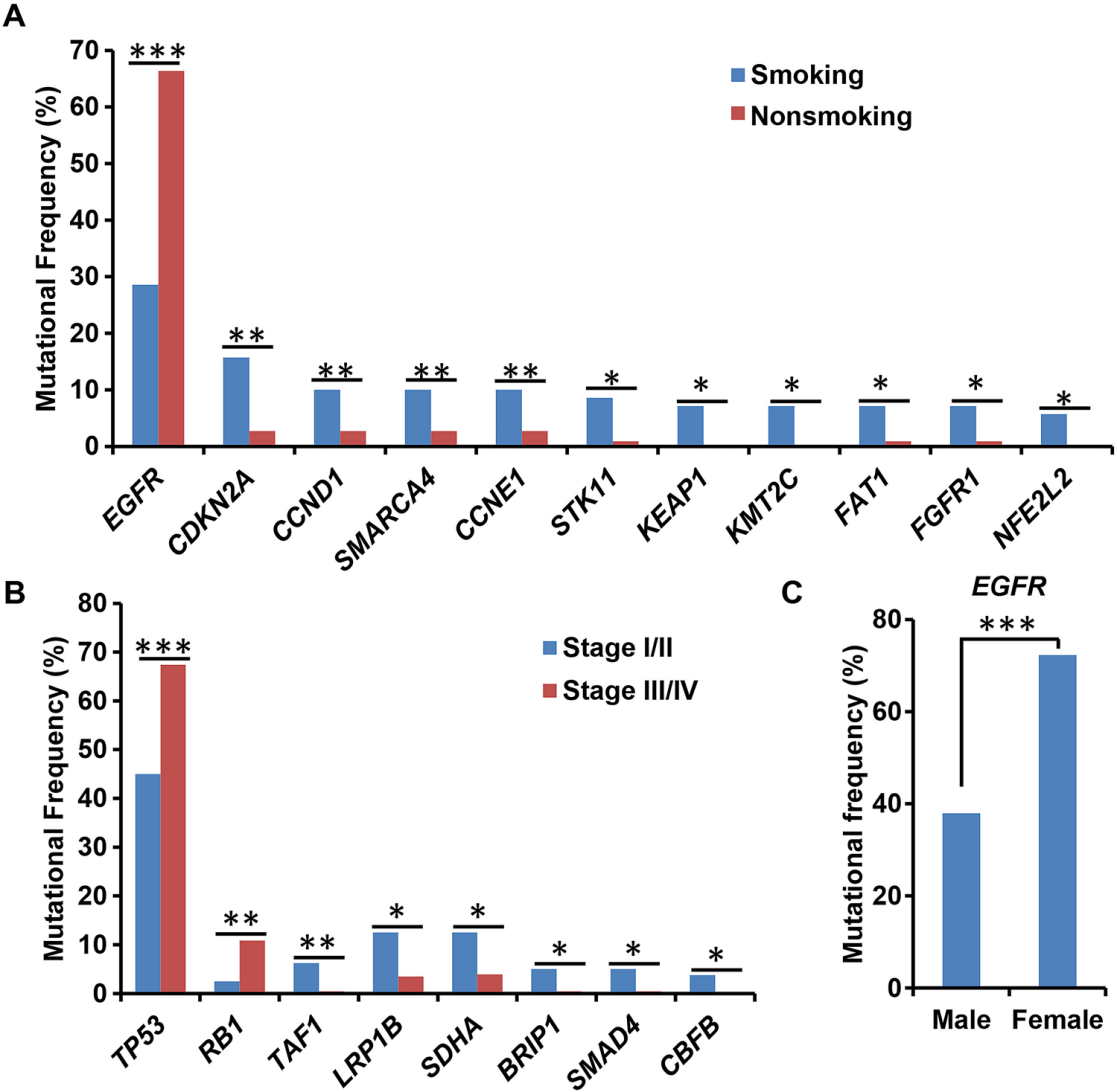
Correlation analysis of mutated genes and clinical characteristics. (**A**) Differences in mutated genes between smoking and nonsmoking patients. (**B**) Correlation between mutated genes and tumor stage. (**C**) Differences in mutational frequency of *EGFR* between male and female lung cancer patients. *P < 0.05, **P < 0.01, and ***P < 0.001.

Based on the smoking status data, we analyzed the correlation between mutated genes and smoking status of patients. The most common mutated genes in smokers included *TP53*, *EGFR*, *KRAS*, *CDKN2A*, *LRP1B*, *ALK*, *BCL2L11*, *KEAP1*, *KMT2C*, *PIK3CA*, and *STK11*. The most common mutated genes in non-smokers were *EGFR*, *TP53*, *RB1*, *SDHA*, *RBM10*, and *TERT* (Table S2). *TP53* and *EGFR* mutations frequently occurred in both smokers and nonsmokers. Based on statistical analysis, the frequency of *EGFR* mutations was significantly higher in nonsmoking than smoking patients (Fig. 7A). The mutation frequencies of *CDKN2A*, *FAT1*, *FGFR1*, *NFE2L2*, *CCNE1*, *CCND1*, *SMARCA4*, *KEAP1*, *KMT2C*, and *STK11* were significantly higher in smokers than in nonsmokers (Fig. 7A).

According to the information on tumor stage, we combined 80 cases of stage I and II into a group, and 230 cases of stage III and IV into another group. Statistical analysis showed that *TP53* and *RB1* mutation frequencies were significantly higher in cases with tumor stages III and IV than in those with tumor stages I and II, while the mutation frequencies of *TAF1*, *LRP1B*, *SDHA*, *CBFB*, *BRIP1*, and *SMAD4* were significantly higher in cases with tumor stages I and II than in those with tumor stages III and IV (Fig. 7B).

We also analyzed the association between mutated genes and sex. The statistical analysis results showed that EGFR mutation was significantly associated with sex (Fig. 7C).

### Correlations between tumor mutation burden and clinical characteristics and mutated genes

**Figure 8:**
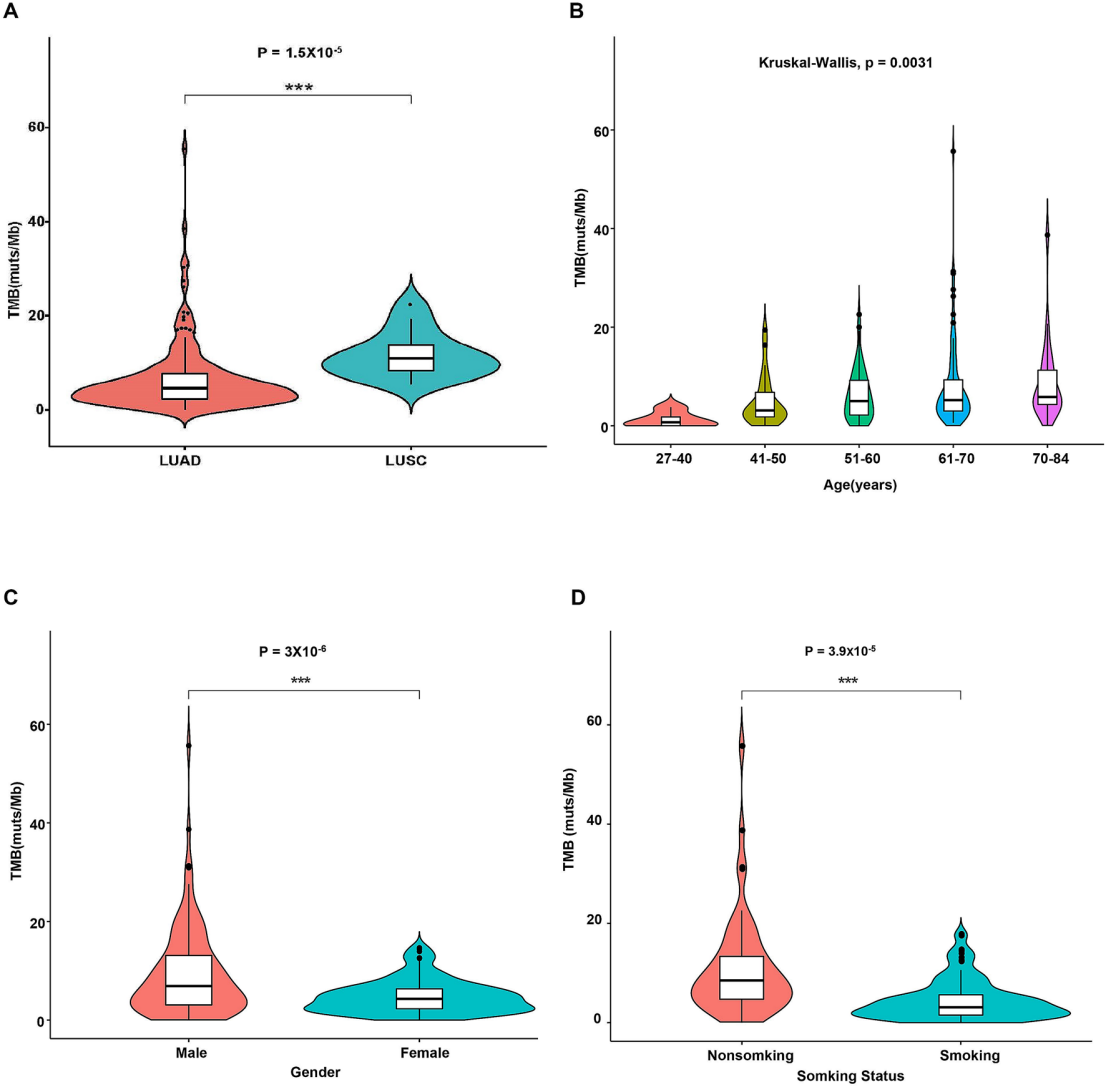
Association between TMB and clinical characteristics. (**A**) Association between TMB and tumor subtypes; (**B**) Association between TMB and age of patients; (**C**) Correlation analysis between TMB and sex; (**D**) Correlation analysis between TMB and smoking status. *LUAD* lung adenocarcinoma, *LUSC* lung squamous cell carcinoma, *LASC* lung adenosquamous cell carcinoma, *SCLC* small cell lung cancer, *TMB* tumor mutational burden.

We measured the available TMB in 216 cases to explore the relationship between TMB and clinical characteristics, and TMB and clinically relevant mutations. The median TMB was 5.0 muts/Mb (range, 0–55.7 muts/Mb) (Table 1). We identified TMB-H in 49 cases (22.7%, 49/216) and TMB-L was identified in 167 cases (77.3%, 167/216). The median TMB in LUSC was higher than that in LUAD. Statistical analysis showed a significant association between TMB and tumor subtype (Fig. 8A). According to the age distribution of patients, we found that the TMB value increased gradually with the increase in age. Statistical analysis showed a positive correlation between age and TMB value in lung cancer (Fig. 8B). In this cohort, we also found that the median TMB was higher in males than in females (7 mutations/Mb vs 4.3 mutations/Mb). The median TMB of smoking and non-smoking patients was 8.5 mutations/Mb and 3.8 mutations/Mb, respectively. Statistical analysis showed a significant association between TMB and sex and smoking status of patients (Fig. 8C,D).

**Figure 9:**
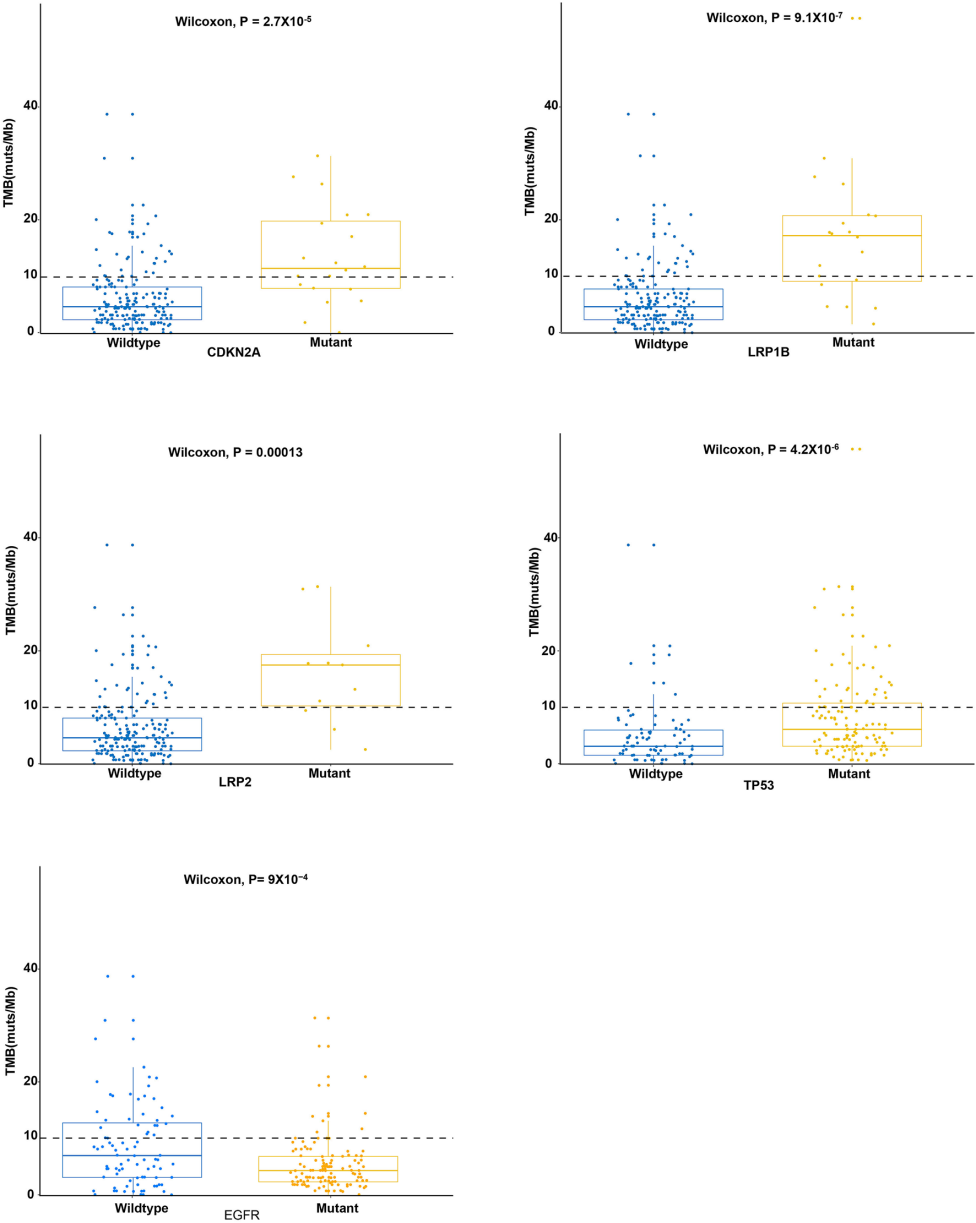
Correlation analysis between TMB and mutated genes. The X-axis shows the gene status and the Y-axis represents TMB values. *TMB* tumor mutational burden.

Based on clinical relevance, we also analyzed TMB-related mutations. For each tested gene, patients were divided into mutant and wild type groups. Statistical analysis showed that mutations in *CDKN2A*, *LRP1B*, *LRP2*, *TP53*, and *EGFR* were significantly associated with TMB. Among these five genes, mutations in *CDKN2A*, *LRP1B*, *LRP2*, and *TP53* were associated with high TMB, while *EGFR* mutations were associated with low TMB (Fig. 9).

### Characterization of *EGFR* mutations in patients resistant to icotinib/gefitinib

In this cohort, 203 patients were harbored *EGFR* mutations, with 77 patients receiving EGFR-TKIs treatment. Of the patients who received this treatment, we followed up 29 patients who treated with icotinib (375 mg/day) or gefitinib (250 mg/day). Among them, 22 patients developed disease progression within 6 months and were considered drug resistant, while 7 benefited from the therapy for more than 6 months and were considered drug sensitive. A total of 55 *EGFR* alterations were detected in these 29 patients, including 8 L858R, 16 T790M, 19 19del, and 12 *EGFR* amplifications. Among the drug-resistant patients, 12 had T790M mutation. The patients who did not have this mutation included 2 patients with L858R mutation (one of them harbored *ERBB2* amplification), 2 patients with *EGFR* amplification, 4 patients with 19del mutation (one of them harbored *ERBB2* amplification), and 2 patients with both 19del mutation and *EGFR* amplification. Among the drug-sensitive patients, 4 had T790M mutation and 3 did not have this mutation (2 carried 19del and 1 carried L858R mutation) (Table 2).

**Table 2:** Lotinib/gefitinib response and EGFR mutations of 29 lung cancer patients.

Cases	Cancer subtype	Drug responses	Tumor stage	EGFR mutation				ERBB2 amplification
1	LUAD	R	IV	T790M			L858R	
2	LUAD	R	III	T790M			L858R	
3	LUAD	R	IV				L858R	
4	LUAD	R	–	T790M			L858R	
5	LUAD	R	IV	T790M			L858R	
6	LUAD	R	III				L858R	yes
7	LUAD	R	IV	T790M	19 exon del	Amplification		
8	LUAD	R	–		19 exon del	Amplification		
9	LUAD	R	IV	T790M	19 exon del	Amplification		
10	SCLC	R	IV		19 exon del	Amplification		
11	LUAD	R	IV	T790M	19 exon del	Amplification		
12	LUAD	R	IV	T790M	19 exon del	Amplification		
13	LUAD	R	IV	T790M	19 exon del	Amplification		
14	LUSC	R	IV	T790M	19 exon del	Amplification		
15	LUAD	R	III		19 exon del			yes
16	LUAD	R	IV	T790M	19 exon del			
17	LUAD	R	–		19 exon del			
18	LUAD	R	–		19 exon del			
19	NSCLC	R	IV		19 exon del			
20	LASC	R	IV			Amplification		
21	SCLC	R	IV			Amplification		
22	LUAD	R	–	T790M		Amplification	L858R	
23	LUAD	S	IV	T790M	19 exon del			
24	NSCLC	S	–	T790M	19 exon del			
25	LUAD	S	IV	T790M	19 exon del			
26	LUAD	S	IV		19 exon del			
27	LUAD	S	–		19 exon del			
28	LUAD	S	IV	T790M	19 exon del	Amplification		
29	LUAD	S	I				L858R	

*LUAD* lung adenocarcinoma, *LUSC* lung squamous cell carcinoma, *LASC* lung adenosquamous cell carcinoma, *SCLC* small cell lung cancer.

**Figure 10:**
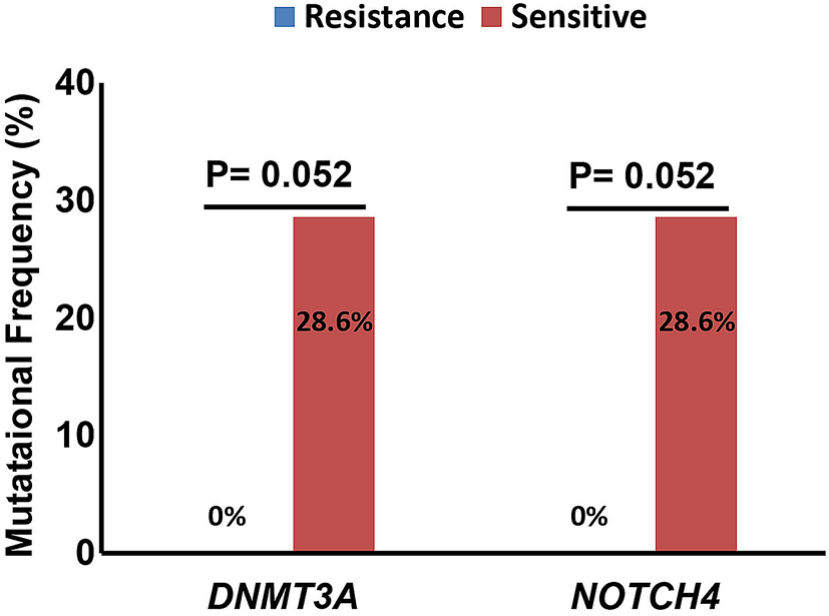
Specific DNMT3A and NOTCH4 mutations in icotinib/gefitinib resistant patients. The X-axis shows the mutated gene and the Y-axis represents mutational frequency of each gene in icotinib/gefitinib resistant and sensitive patients. *TMB* tumor mutational burden.

In addition to the T790M mutation, we found that the proportion of *EGFR* amplification in patients with drug resistance was higher than that in patients with drug sensitivity (40% vs 0%). We also analyzed mutations other than *EGFR* in the followed up patients. We found that *DNMT3A* and *NOTCH4* mutations were lower in the lcotinib/gefitinib-resistant patients than those in the drug-sensitive patients (0% vs 28.6%, P = 0.052, for both) (Fig. 10).

## Discussion

Lung cancer, which is multi-factorial and has various histological subtypes, is one of the most dangerous malignant tumors to human health and life. In recent years, the incidence and mortality rates of lung cancer have increased significantly in many countries^1,30^, with the incidence in women increasing annually^31^. In addition, men are more likely to develop LUSC, while women are more likely to develop LUAD^32^. Regarding risk factors, smoking is one of the most important for lung cancer. Smoking has been reported to be significantly associated with LUSC^33^. Our results also supported that smoking was significantly associated with LUSC and LASC.

A total of 371 lung cancer patients (187 males and 184 females) were included in this study. Most of them were LUAD patients, and the proportion of different sexes in this group was similar. However, in LUSC patients, the proportion of males was higher than females. This might be due to the high proportion of smokers among male patients. Moreover, the incidence rate of lung cancer increases with age^34^. However, the median age of patients in this study was approximately 60 years and there was no significant difference in age distribution among different cancer subtypes, indicating that there was no correlation between tumor subtypes and patient age.

The continuous development of NGS technology facilitates the analysis of the landscape of cancer mutations. LUAD and LUSC are the two major subtypes of lung cancer and previous studies have shown that they have different molecular characteristics. The most common mutations in LUAD were *TP53*, *KRAS*, *KEAP1*, *STK11*, *EGFR*, *NF1*, and *BRAF*^4^; in LUSC, they were *TP53*, *MLL2* (*KMT2D*), *CDKN2A*, *PIK3CA*, *KEAP1* and *NFE2L2*^5^. In contrast to the results of TCGA^4,5^, we detected low mutational frequencies of *STK11*, *NF1*, and *BRAF* were detected in LUAD and low mutational frequency of *KEAP1* in both LUAD and LUSC, indicating the special molecular characteristics of Chinese lung cancer patients. In addition to observing fewer *EGFR* mutations in LUSC than in LUAD patients, which had been reported by Kim et al.^35^, we also identified the different types of mutated genes in Chinese patients, such as *PIK3CA*, *ALK*, *RBM10*, *MET*, *TERT NKX2*-1, *SDHA*, *CDK4,* and *MDM2* in LUAD; and *CCND1*, *FGFR1*, *FGF3*/4/*13*, and *SOX2* in LUSC. Recently, Ding et al. reported that mutations in *PIK3CA*, *FGFR1*, *CCND1*, and *CDKN2* mainly occurred in LUAD, while *TP53* mutations occurred in nearly 90% of LUSC patients^8^. Although we identified *TP53* mutations in 87% of LUSC patients, there was no significant difference in *TP53* mutational frequency between both types of cancer.

In lung cancer, EGFR mutations are frequently co-mutated with *TP53* and *RB1*, while *KRAS* mutations are frequently co-mutated with *STK11*, *KEAP1* and *RBM10*^36^. Similarly, we found co-mutations of *EGFR, TP53*, and *RB1*, and the mutually exclusive mutations of *EGFR* with *STK11*, *KEAP1*, *ALK* and *KRAS* in this study. Concurrent *KRAS* mutations may lead to resistance to osimertinib and MEK inhibitor combined treatment^37^. Mutual exclusive mutations of *EGFR* and *KRAS* may imply the potential opportunity to benefit from TKI-inhibitor therapy.

However, there was no co-mutation of *KRAS* with *STK11* and *KEAP1*. The inactivation of *TP53* and *RB1* is the molecular characterization of SCLC^38^. In this study, 8 out of 11 SCLC patients harbored a co-mutation of *TP53* and *RB1*. All these results support the previous reported molecular features of lung cancer.

Furthermore, we found significantly different mutational frequencies of *NFE2L2* and *KMT2D*. *NFE2L2* is an important gene involved in the regulation of cell response to oxidative damage and chemotherapy^5^. A previous reports suggested that the *NFE2L2* mutation may be a biomarker for the special treatment of LUSC. Another study reported that the *KMT2D* mutation correlates with poor prognosis in NSCLC^39^. In this way, the high frequency of *KMT2D* mutations indicated a poor prognosis of LUSC. However, the small number of LUSC samples is a limitation of this study and more expanded samples are needed to elucidate this association.

Age is an important factor for lung cancer and is often considered when selecting treatment^40^. With the increasing proportion of young lung cancer patients^41^, more attention has been devoted to their diagnosis. Sacher et al. focused on the GAs of young lung cancer patients and identified that mutations in *EGFR*, *ALK*, and *ERBB2* trend to occur in younger NSCLC patients^42^. According to different age groups, Jiang et al. reported that mutations in *EGFR* and *TP53* were associated with age in Chinese NSCLC patients^43^. In contrast to this study, we did not detect an association between age and *TP53* and *EGFR* mutations. However, we detected the correlation between age and *ALK* and *ERBB2* mutations, similar to the results of Sacher et al.^42^, which showed the reliability of our analysis. Furthermore, we identified associations between younger patients and *NRAS* and *KMT2D* mutations, and elderly patients and *RBM10*, *NF1*, *PIK3CA*, *MET*, *PBRM1*, *LRP2*, and *CDKN2B* mutations. These results contribute to the age-associated gene alteration data in lung cancer.

The mutational profile is different in smoking and nonsmoking patients. Mutations in *EGFR*, *ROS1*, and *ALK* mainly occur in nonsmoking patients, while mutations in *KRAS*, *TP53*, *BRAF*, *JAK2*, and *JAK3* mainly occur in smoking patients^44^. However, only *EGFR* mutations were found to be associated with nonsmoking. Although the mutational frequencies of *KRAS* and *TP53* were also higher in smoking than those in nonsmoking patients in this study, the statistical analysis showed no significant difference (17.1% vs. 7.3%, P = 0.052; and 62.9% vs. 52.7%, P = 0.22, respectively). In contrast to previous studies^43,44^, our results revealed a series of smoking-associated genes, including *CDKN2A*, *CCND1*, *SMARCA4*, *CCNE1*, *STK11*, *KEAP1*, *KMT2C*, *FAT1*, *FGFR1*, and *NFE2L2*. This discrepancy may be caused by regional differences among populations.

Previous studies have shown that female patients have a lower risk of cancer progression than male patients^45,46^. Sex-related biomarkers could indicate specific treatment options. Similar to previous studies, we found that the mutational frequency of *EGFR* was significantly higher in female patients than that in male patients^4^. Tumor, lymph node and metastasis (TNM) staging are often used in treatment decisions and prognosis prediction of lung cancer patients^47^. For NSCLC, stages I-II are considered early stages and are normally treated with surgery, while stages III-IV are advanced stages and are normally treated with concurrent chemoradiotherapy^48^. Our results showed a correlation between *TP53* and *RB1* mutations and tumor stages III-IV. *TP53* and *RB1* are important regulators of cell cycle progression. *TP53* is the most common mutated gene in human cancers, and both *TP53* and *RB1* mutations are reported to be associated with poor prognosis of lung cancer patients^49–52^. Our results indicate that these mutations may predict the prognosis of Chinese lung cancer patients.

Moreover, we showed a significant association between early tumor stage and mutations in *TAF1, LRP1B, SDHA, CBFB, BRIP1,* and *SMAD4*. Chen et al. reported that *LRP1B* mutation was associated with better survival in NSCLC patients treated with immunotherapy^53^. Additionally, *SMAD4* expression is associated with survival of patients with lung and pancreatic cancers^54^, while *SDHA* is considered a tumor suppressor gene of paraganglioma^55^. In this study, we first reported the correlation between *SDHA* mutation and tumor stage, indicating its potential predictive value. Although the correlation between *TAF1*, *BRIP1*, and *CBFB* mutations and prognosis has been reported, only have been reported in lung cancer^56–58^. Our results suggest that these genes may be related to prognosis in Chinese lung cancer patients. However, studies with a longer follow-up period are needed to elucidate this relationship.

TMB is a new biomarker that may further guide the selection of checkpoint inhibitors (CPI) for patients^59^. A certain correlation between TMB and clinical characteristics has been reported. Wang et al. reported that the predictive power of TMB in lung cancer immunotherapy response was significantly better for women than for men^60^. In addition, it has been reported that increased TMB is associated with increased age in many types of cancers^61^. Wang et al. reported associations between TMB and smoking history and age of patients with LUAD^62^. In NSCLC patients with TMB-H, non-smokers had a significantly better prognosis compared with smokers^63^. However, there was no difference in TMB values between smoking and nonsmoking SCLC patients^64^. Similar to a previous study, we identified associations between TMB and sex, age, and smoking status. Furthermore, our results showed a significant association between TMB and tumor subtype. However, we also detected a correlation between smoking status and tumor subtype, indicating that the association between tumor subtype and TMB may be caused by the smoking status.

Moreover, TMB is associated with known DNA mismatch repair pathway genes (*MSH2*, *MSH6*, *MLH1*, and *PMS2*) and DNA polymerases (POLE)^61^. In this study, we failed to detect a correlation between TMB and these genes. However, statistical analysis showed the significant association between TMB and mutations in *EGFR*, *TP53*, *LRP1B*, *LRP2*, and *CDKN2A*, suggesting potential biomarkers for the prognosis of Chinese lung cancer patients. Particularly, *TP53* mutation status may be a useful biomarker for predicting the response to immunotherapy in different cancer types^65^. Owada-Ozaki et al. reported that high TMB is associated with poor prognosis in NSCLC^66^. These studies supported our results.

*EGFR*-mutated lung cancer is a special molecular subgroup of lung cancer in which most patients benefit from treatment with EGFR-TKIs^67^. The clinical course of *EGFR* mutant lung cancer is significantly heterogeneous, and acquisition of *EGFR* T790M mutation is the most frequent reason for first- and second-generation EGFR-TKIs^68^. The receptor tyrosine kinase or alternative downstream compounds activate survival tracks such as *MET* amplification, *ERBB2* amplification, and *IGF1R* activation, which are the main EGFR-independent reasons for EGFR-TKIs resistance^69,70^.

Besides 19del and L858R, *EGFR* amplification is also frequently occurs in lung cancer. Recently, Chen et al. reported that an *EGFR*-amplified cervical squamous cell carcinoma patient benefited from afatinib therapy^71^. *EGFR* amplification was also reported to be associated with better OS, PFS, CR, and PR in LUAD patients treated with erlotinib^72^. In this study, 29 patients received the treatment of lcotinib/gefitinib. In addition to the T790M mutation or ERBB2 amplification, we found a higher proportion of *EGFR* amplification in EGFR-TKI-resistant patients than that in EGFR-TKI-sensitive patients. It is important to consider whether EGFR amplification is associated with the rapid development of lcotinib/gefitinib resistance.

However, 6 patients with 19del or L858R mutations also rapidly developed EGFR-TKIs resistance. We found a high mutational proportion of *DNMT3a* and *NOTCH4* in these patients. *DNMT3a* plays an important role in methylation status. The Notch signaling pathway has an important regulatory role in a variety of tumor stem cells. Mutations in *DNMT3a* and *NOTCH4* have been reported to be associated with better prognosis in patients with LUAD and NSCLC, respectively^73,74^. Similarly, the EGFR-TKI sensitivity of patients with these indicates a good prognosis. It is suggested that *DNMT3a* and *NOTCH4* mutations may be potential biomarkers to predict sensitivity to EGFR-TKIs.

In conclusion, we identified the comprehensive genomic features of 371 Chinese lung cancer patients and found that sex and smoking status were significantly associated with lung cancer subtype. Furthermore, we detected that certain gene mutations were associated with age, smoking status, tumor stage, and TMB value. We also suggested a series of biomarkers for potential therapy and prognosis, and indicated that *EGFR* amplification, *DNMT3a* mutation, and *NOTCH4* mutation may be used to predict EGFR-TKI resistance. Together, our research contributes to the comprehensive understanding of lung cancer molecular features and provides evidence for the developing and application of precise therapeutic strategies for Chinese lung cancer patients.

## Supplementary information

Supplementary Information 1.

Supplementary Figure S1.

Supplementary Table S1.

Supplementary Table S2.

## Data Availability

The datasets used and analyzed in this study are available from the corresponding author upon reasonable request.
