# Supplementary material for: Comprehensive genomic profile of Chinese lung cancer patients and mutation characteristics of individuals resistant to icotinib/gefitinib
Source: Sci Rep. 2020 Nov 20;10:20243. doi: 10.1038/s41598-020-76791-y (PMC7679461; doi:10.1038/s41598-020-76791-y)
Supplement: Supplementary file 1 — Supplementary Information 1. [file 41598_2020_76791_MOESM1_ESM.docx]

**Comprehensive genomic profile of Chinese lung cancer patients and mutation characteristics of individuals resistant to icotinib/gefitinib**

Yanhong Shang^1^*, Xiaofang Li^1^, Weiwei Liu^1^, Xiaoliang Shi^2^, Shaohua Yuan^2^, Ran Huo^1^, Guotao Fang^1^, Xiao Han^1^, Jingnan Zhang^1^, Kunjie Wang^1^, Zhengyue Dou^1^, Yan Zhang^3^, Aiming Zang^1^, LinZhang^2^

^1^Department of Medical Oncology, Affiliated Hospital of Hebei University; No.648，Dongfeng East Road, Baoding City, Hebei 071000, P.R. China;

^2^Origimed Co. Ltd, Shanghai 201114, P.R. China

^3^Department of Oncology, The First Hospital of Shijiazhuang City, No.36，Fanxi Road，Chang’an District, Shijiazhuang City, Hebei 050000, P.R. China Abstract

These authors contributed equally: Yanhong Shang, Xiaofang Li and Weiwei Liu

*Correspondence to: Prof. Yanhong Shang

Department of Medical Oncology, Affiliated Hospital of Hebei University, No.648，Dongfeng East Road, Baoding City, Hebei 071000, P.R. China.

E-mail: hdfyzlnk@sina.com

## Supplementary Figures

Figure S1 Distribution of EGFR genomic alterations. The X-axis shows the gene structure and the Y-axis shows the number of mutations in each site. Green represents the Recep_L_domain, red represents the Furin-like domain, blue represents the GF_recep_IV domain, and yellow represents pkinase_Tyr domain.
