# Supplementary figures and images for: Comprehensive genomic profile of Chinese lung cancer patients and mutation characteristics of individuals resistant to icotinib/gefitinib
Source: Sci Rep. 2020 Nov 20;10:20243. doi: 10.1038/s41598-020-76791-y (PMC7679461; doi:10.1038/s41598-020-76791-y)

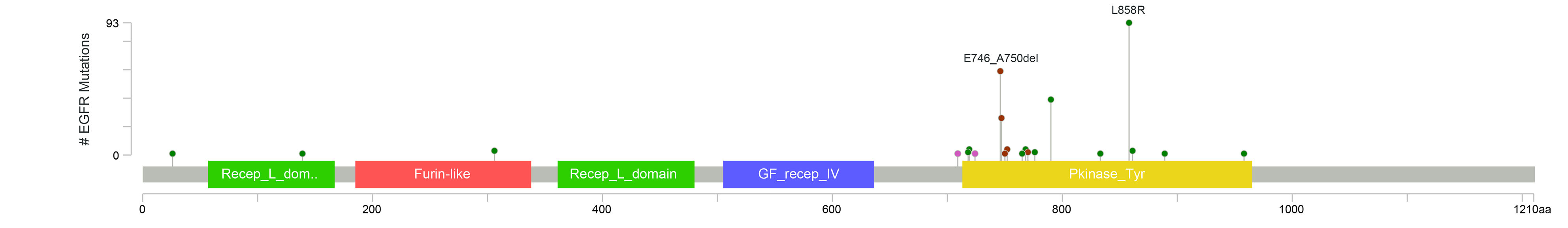

Supplement: Supplementary file 2 — Supplementary Figure S1. [file 41598_2020_76791_MOESM2_ESM.jpg]
