# Supplementary material for: Comprehensive genomic profile of Chinese lung cancer patients and mutation characteristics of individuals resistant to icotinib/gefitinib
Source: Sci Rep. 2020 Nov 20;10:20243. doi: 10.1038/s41598-020-76791-y (PMC7679461; doi:10.1038/s41598-020-76791-y)
Supplement: Supplementary file 3 — Supplementary Table S1. [file 41598_2020_76791_MOESM3_ESM.docx]

Table S1 Total genomic alterations detected in different genes.

| Genes | Fusion/Rearrangement | Gene Amplification | Gene Homozygous Deletion | Substitution/Indel | Truncation | Total | Ratio |
| --- | --- | --- | --- | --- | --- | --- | --- |
| EGFR | 2 | 58 | 0 | 253 | 0 | 313 | 54.7% |
| TP53 | 1 | 0 | 0 | 198 | 54 | 253 | 61.5% |
| KRAS | 0 | 7 | 0 | 35 | 0 | 42 | 11.1% |
| PIK3CA | 0 | 10 | 0 | 30 | 0 | 40 | 9.2% |
| RB1 | 1 | 0 | 2 | 15 | 15 | 33 | 8.6% |
| CDKN2A | 4 | 0 | 11 | 7 | 9 | 31 | 7.8% |
| ALK | 18 | 0 | 0 | 9 | 0 | 27 | 5.9% |
| LRP1B | 1 | 0 | 0 | 21 | 4 | 26 | 5.4% |
| RBM10 | 0 | 0 | 0 | 5 | 21 | 26 | 7.0% |
| BCL2L11 | 0 | 0 | 0 | 22 | 0 | 22 | 5.9% |
| MET | 1 | 10 | 0 | 10 | 0 | 21 | 5.4% |
| SPTA1 | 0 | 0 | 0 | 10 | 11 | 21 | 5.4% |
| TERT | 0 | 16 | 0 | 5 | 0 | 21 | 5.7% |
| BRCA1 | 10 | 0 | 0 | 4 | 6 | 20 | 2.4% |
| APC | 2 | 0 | 0 | 9 | 7 | 18 | 4.3% |
| KMT2C | 1 | 0 | 0 | 12 | 5 | 18 | 3.5% |
| SDHA | 0 | 15 | 0 | 3 | 0 | 18 | 4.9% |
| DNMT3A | 0 | 0 | 0 | 15 | 2 | 17 | 4.3% |
| ERBB2 | 0 | 8 | 0 | 8 | 0 | 16 | 4.3% |
| BRAF | 1 | 3 | 0 | 11 | 0 | 15 | 4.0% |
| NKX2-1 | 0 | 13 | 0 | 0 | 2 | 15 | 4.0% |
| PTEN | 0 | 0 | 0 | 6 | 9 | 15 | 3.8% |
| CTNNB1 | 0 | 0 | 0 | 14 | 0 | 14 | 3.5% |
| MLH1 | 0 | 0 | 0 | 12 | 2 | 14 | 3.5% |
| STK11 | 3 | 0 | 0 | 6 | 5 | 14 | 3.5% |
| KEAP1 | 0 | 0 | 0 | 11 | 2 | 13 | 3.5% |
| MDM2 | 0 | 13 | 0 | 0 | 0 | 13 | 3.2% |
| CDK4 | 0 | 12 | 0 | 0 | 0 | 12 | 3.0% |
| FAT3 | 0 | 0 | 0 | 11 | 1 | 12 | 3.2% |
| FAT4 | 0 | 0 | 0 | 8 | 4 | 12 | 3.2% |
| KMT2D | 1 | 0 | 0 | 3 | 8 | 12 | 3.2% |
| ROS1 | 5 | 0 | 0 | 7 | 0 | 12 | 3.0% |
| CDKN2B | 0 | 0 | 10 | 1 | 0 | 11 | 3.0% |
| FRS2 | 0 | 11 | 0 | 0 | 0 | 11 | 2.7% |
| LRP2 | 0 | 0 | 0 | 10 | 1 | 11 | 3.0% |
| ATM | 1 | 0 | 0 | 5 | 4 | 10 | 2.7% |
| BRCA2 | 2 | 0 | 0 | 5 | 3 | 10 | 2.7% |
| FAM135B | 0 | 3 | 0 | 7 | 0 | 10 | 2.7% |
| MCL1 | 0 | 10 | 0 | 0 | 0 | 10 | 2.7% |
| SMARCA4 | 0 | 0 | 0 | 5 | 5 | 10 | 2.7% |
| CCND1 | 0 | 8 | 0 | 1 | 0 | 9 | 2.4% |
| EP300 | 0 | 0 | 0 | 6 | 3 | 9 | 2.4% |
| NF1 | 1 | 0 | 0 | 5 | 3 | 9 | 2.4% |
| OBSCN | 0 | 0 | 0 | 8 | 1 | 9 | 2.4% |
| SLIT2 | 0 | 0 | 0 | 6 | 3 | 9 | 2.4% |
| CCNE1 | 0 | 8 | 0 | 0 | 0 | 8 | 2.2% |
| CDK12 | 1 | 3 | 0 | 3 | 1 | 8 | 1.9% |
| ERBB4 | 0 | 0 | 0 | 8 | 0 | 8 | 2.2% |
| GNAS | 0 | 3 | 0 | 5 | 0 | 8 | 2.2% |
| MED12 | 0 | 0 | 0 | 3 | 5 | 8 | 1.9% |
| MYC | 0 | 7 | 0 | 1 | 0 | 8 | 2.2% |
| RET | 5 | 0 | 0 | 3 | 0 | 8 | 2.2% |
| SETD2 | 0 | 0 | 0 | 4 | 4 | 8 | 2.2% |
| AKT2 | 0 | 6 | 0 | 1 | 0 | 7 | 1.9% |
| ARID2 | 0 | 1 | 0 | 2 | 4 | 7 | 1.9% |
| ASXL1 | 0 | 0 | 0 | 1 | 6 | 7 | 1.9% |
| BCOR | 1 | 2 | 0 | 3 | 1 | 7 | 1.3% |
| CARD11 | 0 | 4 | 0 | 2 | 1 | 7 | 1.9% |
| FAT1 | 0 | 0 | 0 | 5 | 2 | 7 | 1.9% |
| FGF19 | 0 | 7 | 0 | 0 | 0 | 7 | 1.9% |
| FGF3 | 0 | 6 | 0 | 1 | 0 | 7 | 1.9% |
| FGFR1 | 0 | 6 | 0 | 1 | 0 | 7 | 1.9% |
| GLI1 | 0 | 5 | 0 | 2 | 0 | 7 | 1.9% |
| MSH6 | 0 | 0 | 0 | 6 | 1 | 7 | 1.9% |
| NFE2L2 | 0 | 1 | 0 | 6 | 0 | 7 | 1.9% |
| NTRK1 | 2 | 2 | 0 | 3 | 0 | 7 | 1.6% |
| NTRK3 | 1 | 0 | 0 | 6 | 0 | 7 | 1.9% |
| PALB2 | 5 | 0 | 0 | 1 | 1 | 7 | 1.3% |
| PDGFRA | 0 | 1 | 0 | 6 | 0 | 7 | 1.9% |
| SMAD4 | 0 | 0 | 0 | 6 | 1 | 7 | 1.6% |
| SPINK1 | 0 | 1 | 0 | 6 | 0 | 7 | 1.9% |
| ZNF217 | 0 | 5 | 0 | 2 | 0 | 7 | 1.9% |
| ARID1A | 0 | 0 | 0 | 1 | 5 | 6 | 1.6% |
| FGF4 | 0 | 6 | 0 | 0 | 0 | 6 | 1.6% |
| H3F3A | 0 | 4 | 0 | 2 | 0 | 6 | 1.6% |
| HMGA2 | 0 | 6 | 0 | 0 | 0 | 6 | 1.6% |
| IL7R | 0 | 4 | 0 | 2 | 0 | 6 | 1.6% |
| KIT | 0 | 2 | 0 | 4 | 0 | 6 | 1.3% |
| MUC16 | 0 | 0 | 0 | 6 | 0 | 6 | 1.6% |
| NOTCH3 | 0 | 0 | 0 | 6 | 0 | 6 | 1.3% |
| NOTCH4 | 1 | 1 | 0 | 2 | 2 | 6 | 1.6% |
| POLE | 1 | 0 | 0 | 4 | 1 | 6 | 1.6% |
| RICTOR | 0 | 5 | 0 | 1 | 0 | 6 | 1.6% |
| SPEN | 0 | 0 | 0 | 4 | 2 | 6 | 1.6% |
| SRMS | 0 | 5 | 0 | 1 | 0 | 6 | 1.6% |
| STK24 | 0 | 5 | 0 | 1 | 0 | 6 | 1.6% |
| TET2 | 0 | 0 | 0 | 6 | 0 | 6 | 1.6% |
| TRIO | 0 | 6 | 0 | 0 | 0 | 6 | 1.6% |
| TSHR | 0 | 0 | 0 | 6 | 0 | 6 | 1.6% |
| AR | 0 | 0 | 0 | 5 | 0 | 5 | 1.3% |
| AXL | 1 | 2 | 0 | 2 | 0 | 5 | 1.3% |
| BCL6 | 0 | 5 | 0 | 0 | 0 | 5 | 1.3% |
| BRIP1 | 1 | 0 | 0 | 3 | 1 | 5 | 1.3% |
| CFTR | 0 | 3 | 0 | 1 | 1 | 5 | 1.3% |
| CIC | 0 | 0 | 0 | 2 | 3 | 5 | 1.3% |
| CREBBP | 0 | 0 | 0 | 4 | 1 | 5 | 1.3% |
| EPHB1 | 0 | 0 | 0 | 4 | 1 | 5 | 1.3% |
| GLI3 | 0 | 3 | 0 | 2 | 0 | 5 | 1.3% |
| GRM3 | 0 | 1 | 0 | 4 | 0 | 5 | 1.3% |
| HDAC9 | 0 | 0 | 0 | 5 | 0 | 5 | 1.3% |
| JAK2 | 0 | 1 | 0 | 3 | 1 | 5 | 1.3% |
| KLHL6 | 0 | 4 | 0 | 1 | 0 | 5 | 1.3% |
| MACC1 | 0 | 3 | 0 | 2 | 0 | 5 | 1.3% |
| NCOR1 | 1 | 0 | 0 | 1 | 3 | 5 | 1.3% |
| PTK6 | 0 | 5 | 0 | 0 | 0 | 5 | 1.3% |
| RAC1 | 0 | 4 | 0 | 1 | 0 | 5 | 1.3% |
| RUNX1T1 | 0 | 0 | 0 | 4 | 1 | 5 | 1.3% |
| SOX2 | 0 | 5 | 0 | 0 | 0 | 5 | 1.3% |
| TAF1 | 0 | 1 | 0 | 4 | 0 | 5 | 1.3% |
| TSC1 | 2 | 0 | 0 | 1 | 2 | 5 | 1.1% |
| TSC2 | 3 | 0 | 0 | 2 | 0 | 5 | 1.3% |
| ADGRA2 | 0 | 4 | 0 | 0 | 0 | 4 | 1.1% |
| ARFRP1 | 0 | 4 | 0 | 0 | 0 | 4 | 1.1% |
| ATRX | 0 | 0 | 0 | 2 | 2 | 4 | 1.1% |
| AURKA | 0 | 2 | 0 | 2 | 0 | 4 | 1.1% |
| CAMTA1 | 0 | 0 | 0 | 3 | 1 | 4 | 1.1% |
| CDH1 | 0 | 0 | 0 | 4 | 0 | 4 | 1.1% |
| CDK6 | 0 | 4 | 0 | 0 | 0 | 4 | 1.1% |
| CUL3 | 0 | 0 | 0 | 1 | 3 | 4 | 1.1% |
| DDR2 | 0 | 1 | 0 | 3 | 0 | 4 | 1.1% |
| EPHA3 | 0 | 1 | 1 | 0 | 2 | 4 | 1.1% |
| FBXW7 | 0 | 0 | 0 | 3 | 1 | 4 | 1.1% |
| GRIN2A | 0 | 0 | 0 | 4 | 0 | 4 | 1.1% |
| IKZF1 | 0 | 1 | 0 | 2 | 1 | 4 | 1.1% |
| INHBA | 0 | 2 | 0 | 2 | 0 | 4 | 1.1% |
| KDM5C | 1 | 1 | 0 | 1 | 1 | 4 | 1.1% |
| LZTR1 | 0 | 0 | 0 | 0 | 4 | 4 | 1.1% |
| MAP3K13 | 0 | 4 | 0 | 0 | 0 | 4 | 1.1% |
| MTOR | 0 | 0 | 0 | 4 | 0 | 4 | 1.1% |
| NOTCH1 | 0 | 0 | 0 | 2 | 2 | 4 | 1.1% |
| NRG3 | 0 | 0 | 0 | 4 | 0 | 4 | 1.1% |
| PBRM1 | 0 | 0 | 0 | 2 | 2 | 4 | 1.1% |
| PMS2 | 1 | 0 | 0 | 1 | 2 | 4 | 1.1% |
| RPTOR | 0 | 2 | 0 | 2 | 0 | 4 | 1.1% |
| WT1 | 0 | 1 | 0 | 3 | 0 | 4 | 1.1% |
| ABCB1 | 0 | 0 | 0 | 1 | 2 | 3 | 0.8% |
| AMER1 | 0 | 0 | 0 | 1 | 2 | 3 | 0.8% |
| ARHGEF17 | 0 | 3 | 0 | 0 | 0 | 3 | 0.8% |
| ARID1B | 0 | 0 | 0 | 2 | 1 | 3 | 0.8% |
| AXIN1 | 0 | 0 | 0 | 3 | 0 | 3 | 0.8% |
| BCR | 0 | 0 | 0 | 3 | 0 | 3 | 0.8% |
| CBFB | 0 | 0 | 0 | 2 | 1 | 3 | 0.8% |
| CCND3 | 0 | 2 | 0 | 1 | 0 | 3 | 0.8% |
| CHD2 | 0 | 0 | 0 | 3 | 0 | 3 | 0.8% |
| COL1A1 | 1 | 0 | 0 | 2 | 0 | 3 | 0.8% |
| CRLF2 | 0 | 3 | 0 | 0 | 0 | 3 | 0.8% |
| EMSY | 0 | 3 | 0 | 0 | 0 | 3 | 0.8% |
| EPHA5 | 0 | 0 | 0 | 2 | 1 | 3 | 0.8% |
| ERRFI1 | 0 | 0 | 0 | 0 | 3 | 3 | 0.8% |
| ESR1 | 0 | 0 | 0 | 3 | 0 | 3 | 0.8% |
| ETV1 | 0 | 1 | 0 | 2 | 0 | 3 | 0.8% |
| ETV6 | 1 | 0 | 0 | 1 | 1 | 3 | 0.8% |
| FANCA | 0 | 0 | 0 | 2 | 1 | 3 | 0.8% |
| FANCD2 | 0 | 0 | 0 | 3 | 0 | 3 | 0.8% |
| FGF10 | 0 | 3 | 0 | 0 | 0 | 3 | 0.8% |
| FGFR3 | 0 | 0 | 0 | 3 | 0 | 3 | 0.8% |
| FLT3 | 0 | 0 | 0 | 3 | 0 | 3 | 0.8% |
| FLT4 | 0 | 1 | 0 | 2 | 0 | 3 | 0.8% |
| FUS | 0 | 1 | 0 | 1 | 1 | 3 | 0.8% |
| GATA6 | 0 | 0 | 0 | 3 | 0 | 3 | 0.8% |
| HGF | 0 | 1 | 0 | 2 | 0 | 3 | 0.8% |
| INPP4B | 0 | 0 | 0 | 3 | 0 | 3 | 0.8% |
| KEL | 0 | 0 | 0 | 2 | 1 | 3 | 0.8% |
| LRP1 | 1 | 0 | 0 | 2 | 0 | 3 | 0.5% |
| MEN1 | 0 | 0 | 0 | 2 | 1 | 3 | 0.8% |
| MST1R | 0 | 0 | 0 | 3 | 0 | 3 | 0.8% |
| MYCN | 0 | 0 | 0 | 3 | 0 | 3 | 0.8% |
| NF2 | 0 | 0 | 0 | 2 | 1 | 3 | 0.8% |
| NOTCH2 | 0 | 0 | 0 | 2 | 1 | 3 | 0.8% |
| NRAS | 0 | 0 | 0 | 3 | 0 | 3 | 0.8% |
| PARP1 | 1 | 0 | 0 | 2 | 0 | 3 | 0.8% |
| PDGFB | 2 | 0 | 0 | 1 | 0 | 3 | 0.8% |
| PIK3C2B | 0 | 1 | 0 | 2 | 0 | 3 | 0.8% |
| PIK3CB | 0 | 1 | 0 | 2 | 0 | 3 | 0.8% |
| PRSS8 | 1 | 0 | 0 | 1 | 1 | 3 | 0.5% |
| RAD21 | 0 | 3 | 0 | 0 | 0 | 3 | 0.8% |
| RAD50 | 0 | 0 | 0 | 1 | 2 | 3 | 0.8% |
| RANBP2 | 0 | 0 | 0 | 1 | 2 | 3 | 0.8% |
| REL | 0 | 1 | 0 | 2 | 0 | 3 | 0.8% |
| RIT1 | 0 | 1 | 0 | 2 | 0 | 3 | 0.8% |
| RUNX1 | 0 | 0 | 0 | 1 | 2 | 3 | 0.8% |
| SETBP1 | 0 | 0 | 0 | 3 | 0 | 3 | 0.8% |
| TBX3 | 0 | 0 | 0 | 3 | 0 | 3 | 0.8% |
| TET1 | 0 | 0 | 0 | 2 | 1 | 3 | 0.8% |
| USP6 | 0 | 0 | 0 | 3 | 0 | 3 | 0.8% |
| VEGFA | 0 | 2 | 0 | 1 | 0 | 3 | 0.8% |
| ZNF703 | 0 | 2 | 0 | 1 | 0 | 3 | 0.8% |
| ABL1 | 0 | 0 | 0 | 2 | 0 | 2 | 0.5% |
| APOBEC3B | 0 | 2 | 0 | 0 | 0 | 2 | 0.5% |
| ARAF | 0 | 1 | 0 | 1 | 0 | 2 | 0.5% |
| ARHGEF25 | 0 | 1 | 0 | 1 | 0 | 2 | 0.5% |
| ATR | 0 | 0 | 0 | 2 | 0 | 2 | 0.5% |
| B2M | 1 | 0 | 0 | 1 | 0 | 2 | 0.5% |
| BARD1 | 0 | 0 | 0 | 2 | 0 | 2 | 0.5% |
| BRD4 | 0 | 0 | 0 | 2 | 0 | 2 | 0.5% |
| CD1A | 0 | 0 | 0 | 2 | 0 | 2 | 0.5% |
| CEBPA | 0 | 0 | 0 | 2 | 0 | 2 | 0.5% |
| CHD4 | 0 | 0 | 0 | 2 | 0 | 2 | 0.5% |
| CHEK1 | 0 | 1 | 0 | 1 | 0 | 2 | 0.5% |
| CRBN | 0 | 0 | 0 | 1 | 1 | 2 | 0.5% |
| CSNK1A1 | 0 | 0 | 0 | 2 | 0 | 2 | 0.5% |
| DDR1 | 0 | 1 | 0 | 1 | 0 | 2 | 0.5% |
| DICER1 | 0 | 0 | 0 | 1 | 1 | 2 | 0.5% |
| DNMT3B | 0 | 1 | 0 | 1 | 0 | 2 | 0.5% |
| EPHA6 | 0 | 0 | 0 | 2 | 0 | 2 | 0.5% |
| EPHA7 | 0 | 0 | 0 | 2 | 0 | 2 | 0.5% |
| ERBB3 | 0 | 1 | 0 | 1 | 0 | 2 | 0.5% |
| ERCC1 | 0 | 2 | 0 | 0 | 0 | 2 | 0.5% |
| ETV5 | 0 | 1 | 0 | 1 | 0 | 2 | 0.5% |
| EZH2 | 0 | 1 | 0 | 1 | 0 | 2 | 0.5% |
| FANCC | 0 | 0 | 0 | 2 | 0 | 2 | 0.3% |
| FGF14 | 0 | 1 | 0 | 1 | 0 | 2 | 0.5% |
| FGFR2 | 0 | 0 | 0 | 2 | 0 | 2 | 0.5% |
| FLCN | 0 | 0 | 0 | 0 | 2 | 2 | 0.5% |
| FUBP1 | 0 | 1 | 0 | 1 | 0 | 2 | 0.5% |
| GATA3 | 0 | 1 | 0 | 1 | 0 | 2 | 0.5% |
| IRF4 | 0 | 0 | 0 | 2 | 0 | 2 | 0.5% |
| IRS2 | 0 | 0 | 0 | 2 | 0 | 2 | 0.5% |
| KAT6A | 0 | 2 | 0 | 0 | 0 | 2 | 0.5% |
| KDM5A | 0 | 1 | 0 | 1 | 0 | 2 | 0.5% |
| KDM6A | 0 | 0 | 0 | 1 | 1 | 2 | 0.5% |
| LIMK1 | 0 | 0 | 0 | 2 | 0 | 2 | 0.5% |
| MAGI2 | 0 | 0 | 0 | 2 | 0 | 2 | 0.5% |
| MAP2K4 | 0 | 0 | 0 | 2 | 0 | 2 | 0.5% |
| MPL | 0 | 1 | 0 | 1 | 0 | 2 | 0.5% |
| MSH2 | 1 | 0 | 0 | 1 | 0 | 2 | 0.5% |
| MTAP | 0 | 0 | 2 | 0 | 0 | 2 | 0.5% |
| MYCL | 0 | 2 | 0 | 0 | 0 | 2 | 0.5% |
| NCOA2 | 0 | 0 | 0 | 1 | 1 | 2 | 0.5% |
| NET1 | 0 | 2 | 0 | 0 | 0 | 2 | 0.5% |
| NPM1 | 0 | 2 | 0 | 0 | 0 | 2 | 0.5% |
| NSD1 | 0 | 0 | 0 | 1 | 1 | 2 | 0.5% |
| PAK1 | 0 | 2 | 0 | 0 | 0 | 2 | 0.5% |
| PAK3 | 0 | 1 | 0 | 1 | 0 | 2 | 0.5% |
| PARP4 | 1 | 0 | 0 | 1 | 0 | 2 | 0.5% |
| PAX5 | 1 | 0 | 0 | 1 | 0 | 2 | 0.5% |
| PDGFRB | 0 | 0 | 0 | 2 | 0 | 2 | 0.5% |
| PIK3CD | 0 | 0 | 0 | 2 | 0 | 2 | 0.5% |
| PLCG2 | 0 | 0 | 0 | 2 | 0 | 2 | 0.5% |
| POLB | 0 | 2 | 0 | 0 | 0 | 2 | 0.5% |
| PPP2R1A | 0 | 0 | 0 | 1 | 1 | 2 | 0.5% |
| PREX2 | 0 | 0 | 0 | 2 | 0 | 2 | 0.5% |
| PRKCI | 0 | 2 | 0 | 0 | 0 | 2 | 0.5% |
| PRKDC | 0 | 0 | 0 | 0 | 2 | 2 | 0.5% |
| PTCH1 | 0 | 0 | 0 | 2 | 0 | 2 | 0.5% |
| PTK2 | 0 | 2 | 0 | 0 | 0 | 2 | 0.5% |
| PTPN11 | 0 | 0 | 0 | 2 | 0 | 2 | 0.5% |
| RAD52 | 0 | 1 | 0 | 1 | 0 | 2 | 0.5% |
| RAD54B | 0 | 1 | 0 | 0 | 1 | 2 | 0.5% |
| RARA | 0 | 1 | 0 | 1 | 0 | 2 | 0.5% |
| RELA | 0 | 1 | 0 | 1 | 0 | 2 | 0.5% |
| ROCK1 | 0 | 1 | 0 | 1 | 0 | 2 | 0.5% |
| RSPO2 | 0 | 2 | 0 | 0 | 0 | 2 | 0.5% |
| SDHB | 0 | 0 | 0 | 0 | 2 | 2 | 0.5% |
| SF3B1 | 0 | 0 | 0 | 2 | 0 | 2 | 0.5% |
| SKP2 | 0 | 2 | 0 | 0 | 0 | 2 | 0.5% |
| SMARCB1 | 0 | 0 | 0 | 1 | 1 | 2 | 0.5% |
| SRSF2 | 0 | 0 | 0 | 2 | 0 | 2 | 0.5% |
| STAG2 | 0 | 0 | 0 | 0 | 2 | 2 | 0.5% |
| STAT3 | 0 | 0 | 0 | 2 | 0 | 2 | 0.5% |
| TGFBR2 | 0 | 0 | 0 | 1 | 1 | 2 | 0.5% |
| TIE1 | 0 | 2 | 0 | 0 | 0 | 2 | 0.5% |
| TNFAIP3 | 0 | 0 | 0 | 2 | 0 | 2 | 0.5% |
| TP63 | 0 | 2 | 0 | 0 | 0 | 2 | 0.5% |
| TSPAN31 | 0 | 2 | 0 | 0 | 0 | 2 | 0.5% |
| XIAP | 0 | 1 | 0 | 1 | 0 | 2 | 0.5% |
| ZNF750 | 0 | 0 | 0 | 1 | 1 | 2 | 0.5% |
| ADAM29 | 0 | 0 | 0 | 1 | 0 | 1 | 0.3% |
| AKT1 | 0 | 0 | 0 | 1 | 0 | 1 | 0.3% |
| AKT3 | 0 | 0 | 0 | 1 | 0 | 1 | 0.3% |
| ARHGDIA | 0 | 0 | 0 | 1 | 0 | 1 | 0.3% |
| ARHGEF10 | 0 | 0 | 1 | 0 | 0 | 1 | 0.3% |
| ARHGEF3 | 0 | 0 | 0 | 1 | 0 | 1 | 0.3% |
| BCL2L2 | 0 | 1 | 0 | 0 | 0 | 1 | 0.3% |
| BCORL1 | 0 | 0 | 0 | 0 | 1 | 1 | 0.3% |
| BIRC3 | 0 | 1 | 0 | 0 | 0 | 1 | 0.3% |
| BLM | 0 | 0 | 0 | 1 | 0 | 1 | 0.3% |
| BTK | 0 | 1 | 0 | 0 | 0 | 1 | 0.3% |
| CALR | 0 | 1 | 0 | 0 | 0 | 1 | 0.3% |
| CASP8 | 0 | 0 | 0 | 1 | 0 | 1 | 0.3% |
| CD1E | 0 | 0 | 0 | 1 | 0 | 1 | 0.3% |
| CD22 | 0 | 1 | 0 | 0 | 0 | 1 | 0.3% |
| CD274 | 0 | 1 | 0 | 0 | 0 | 1 | 0.3% |
| CD36 | 0 | 1 | 0 | 0 | 0 | 1 | 0.3% |
| CD79A | 0 | 1 | 0 | 0 | 0 | 1 | 0.3% |
| CDKN1A | 0 | 0 | 0 | 1 | 0 | 1 | 0.3% |
| CHEK2 | 0 | 0 | 0 | 1 | 0 | 1 | 0.3% |
| CREB3L2 | 0 | 1 | 0 | 0 | 0 | 1 | 0.3% |
| CRKL | 0 | 1 | 0 | 0 | 0 | 1 | 0.3% |
| CUL4A | 0 | 1 | 0 | 0 | 0 | 1 | 0.3% |
| CYLD | 0 | 0 | 1 | 0 | 0 | 1 | 0.3% |
| CYP2D6 | 0 | 0 | 0 | 1 | 0 | 1 | 0.3% |
| DAXX | 1 | 0 | 0 | 0 | 0 | 1 | 0.3% |
| DIS3 | 0 | 0 | 0 | 1 | 0 | 1 | 0.3% |
| DOT1L | 0 | 0 | 0 | 1 | 0 | 1 | 0.3% |
| DPYD | 0 | 0 | 0 | 1 | 0 | 1 | 0.3% |
| EPHA8 | 0 | 0 | 0 | 1 | 0 | 1 | 0.3% |
| EPHB4 | 0 | 1 | 0 | 0 | 0 | 1 | 0.3% |
| ERG | 0 | 0 | 0 | 1 | 0 | 1 | 0.3% |
| ETV4 | 0 | 0 | 0 | 1 | 0 | 1 | 0.3% |
| EWSR1 | 0 | 0 | 0 | 1 | 0 | 1 | 0.3% |
| EZR | 0 | 1 | 0 | 0 | 0 | 1 | 0.3% |
| FAM46C | 0 | 0 | 0 | 1 | 0 | 1 | 0.3% |
| FANCF | 0 | 0 | 0 | 1 | 0 | 1 | 0.3% |
| FANCG | 0 | 0 | 0 | 1 | 0 | 1 | 0.3% |
| FANCL | 0 | 0 | 0 | 0 | 1 | 1 | 0.3% |
| FANCM | 0 | 0 | 0 | 0 | 1 | 1 | 0.3% |
| FEV | 0 | 0 | 0 | 1 | 0 | 1 | 0.3% |
| FGF1 | 0 | 1 | 0 | 0 | 0 | 1 | 0.3% |
| FGF5 | 0 | 0 | 0 | 1 | 0 | 1 | 0.3% |
| FH | 0 | 0 | 0 | 1 | 0 | 1 | 0.3% |
| FLI1 | 0 | 1 | 0 | 0 | 0 | 1 | 0.3% |
| FOS | 0 | 0 | 0 | 1 | 0 | 1 | 0.3% |
| FOXL2 | 0 | 0 | 0 | 1 | 0 | 1 | 0.3% |
| GABRA6 | 0 | 0 | 0 | 1 | 0 | 1 | 0.3% |
| GLI2 | 0 | 0 | 0 | 1 | 0 | 1 | 0.3% |
| HCK | 0 | 0 | 0 | 1 | 0 | 1 | 0.3% |
| HSD3B1 | 0 | 0 | 0 | 1 | 0 | 1 | 0.3% |
| HSP90AA1 | 0 | 0 | 0 | 1 | 0 | 1 | 0.3% |
| IGF1R | 0 | 1 | 0 | 0 | 0 | 1 | 0.3% |
| IGF2 | 0 | 0 | 0 | 1 | 0 | 1 | 0.3% |
| ITK | 0 | 0 | 0 | 1 | 0 | 1 | 0.3% |
| JAK3 | 0 | 0 | 0 | 1 | 0 | 1 | 0.3% |
| KDR | 0 | 0 | 0 | 1 | 0 | 1 | 0.3% |
| LCK | 0 | 0 | 0 | 1 | 0 | 1 | 0.3% |
| MAP2K1 | 0 | 0 | 0 | 1 | 0 | 1 | 0.3% |
| MAP3K1 | 0 | 0 | 0 | 1 | 0 | 1 | 0.3% |
| MAP4K5 | 0 | 1 | 0 | 0 | 0 | 1 | 0.3% |
| MGMT | 0 | 0 | 0 | 1 | 0 | 1 | 0.3% |
| MR1 | 0 | 0 | 0 | 1 | 0 | 1 | 0.3% |
| MUTYH | 0 | 0 | 0 | 1 | 0 | 1 | 0.3% |
| MYD88 | 0 | 0 | 0 | 1 | 0 | 1 | 0.3% |
| MYOD1 | 0 | 1 | 0 | 0 | 0 | 1 | 0.3% |
| NFIB | 0 | 1 | 0 | 0 | 0 | 1 | 0.3% |
| NR4A3 | 0 | 0 | 0 | 1 | 0 | 1 | 0.3% |
| NSD2 | 0 | 1 | 0 | 0 | 0 | 1 | 0.3% |
| PARP3 | 0 | 0 | 0 | 0 | 1 | 1 | 0.3% |
| PBX1 | 0 | 0 | 0 | 1 | 0 | 1 | 0.3% |
| PDCD1LG2 | 0 | 1 | 0 | 0 | 0 | 1 | 0.3% |
| PDK1 | 0 | 0 | 0 | 1 | 0 | 1 | 0.3% |
| PIK3C2G | 0 | 0 | 0 | 1 | 0 | 1 | 0.3% |
| PIK3C3 | 0 | 0 | 0 | 1 | 0 | 1 | 0.3% |
| PIK3CG | 0 | 0 | 0 | 1 | 0 | 1 | 0.3% |
| PIK3R2 | 0 | 0 | 0 | 1 | 0 | 1 | 0.3% |
| PKD2 | 0 | 0 | 0 | 1 | 0 | 1 | 0.3% |
| PRSS1 | 0 | 0 | 0 | 1 | 0 | 1 | 0.3% |
| QKI | 0 | 0 | 0 | 1 | 0 | 1 | 0.3% |
| RAD51 | 1 | 0 | 0 | 0 | 0 | 1 | 0.3% |
| RAD51B | 0 | 0 | 0 | 1 | 0 | 1 | 0.3% |
| RAD54L | 0 | 0 | 0 | 1 | 0 | 1 | 0.3% |
| RAF1 | 0 | 0 | 0 | 1 | 0 | 1 | 0.3% |
| RECQL4 | 0 | 1 | 0 | 0 | 0 | 1 | 0.3% |
| REV3L | 0 | 0 | 0 | 1 | 0 | 1 | 0.3% |
| RHOA | 0 | 0 | 0 | 1 | 0 | 1 | 0.3% |
| RNF43 | 0 | 0 | 0 | 0 | 1 | 1 | 0.3% |
| SDC4 | 0 | 1 | 0 | 0 | 0 | 1 | 0.3% |
| SMAD3 | 0 | 0 | 0 | 1 | 0 | 1 | 0.3% |
| SMO | 0 | 0 | 0 | 1 | 0 | 1 | 0.3% |
| SND1 | 0 | 0 | 0 | 1 | 0 | 1 | 0.3% |
| SPOP | 0 | 0 | 0 | 1 | 0 | 1 | 0.3% |
| SRC | 0 | 1 | 0 | 0 | 0 | 1 | 0.3% |
| SRGAP1 | 0 | 0 | 0 | 1 | 0 | 1 | 0.3% |
| SS18 | 0 | 0 | 0 | 1 | 0 | 1 | 0.3% |
| STAT6 | 0 | 1 | 0 | 0 | 0 | 1 | 0.3% |
| SUFU | 0 | 0 | 0 | 1 | 0 | 1 | 0.3% |
| TCF7L2 | 0 | 0 | 0 | 0 | 1 | 1 | 0.3% |
| TFE3 | 0 | 1 | 0 | 0 | 0 | 1 | 0.3% |
| TFEB | 0 | 1 | 0 | 0 | 0 | 1 | 0.3% |
| TGFBR1 | 0 | 0 | 0 | 1 | 0 | 1 | 0.3% |
| TMPRSS2 | 0 | 1 | 0 | 0 | 0 | 1 | 0.3% |
| TNFRSF14 | 0 | 0 | 0 | 1 | 0 | 1 | 0.3% |
| TNFSF11 | 0 | 0 | 0 | 1 | 0 | 1 | 0.3% |
| TNFSF13B | 0 | 1 | 0 | 0 | 0 | 1 | 0.3% |
| TNK2 | 0 | 1 | 0 | 0 | 0 | 1 | 0.3% |
| TOP1 | 0 | 1 | 0 | 0 | 0 | 1 | 0.3% |
| TOP2A | 0 | 1 | 0 | 0 | 0 | 1 | 0.3% |
| TRAF7 | 0 | 0 | 0 | 0 | 1 | 1 | 0.3% |
| TYRO3 | 0 | 0 | 0 | 1 | 0 | 1 | 0.3% |
| U2AF1 | 0 | 0 | 0 | 1 | 0 | 1 | 0.3% |
| VGLL3 | 0 | 0 | 0 | 0 | 1 | 1 | 0.3% |
| VHL | 0 | 0 | 0 | 1 | 0 | 1 | 0.3% |
| WEE1 | 0 | 0 | 0 | 1 | 0 | 1 | 0.3% |
| WRN | 0 | 0 | 0 | 1 | 0 | 1 | 0.3% |
| XPO1 | 0 | 1 | 0 | 0 | 0 | 1 | 0.3% |
| XRCC3 | 1 | 0 | 0 | 0 | 0 | 1 | 0.3% |
| YAP1 | 0 | 1 | 0 | 0 | 0 | 1 | 0.3% |
| YES1 | 0 | 1 | 0 | 0 | 0 | 1 | 0.3% |
