# Supplementary material for: Comprehensive genomic profile of Chinese lung cancer patients and mutation characteristics of individuals resistant to icotinib/gefitinib
Source: Sci Rep. 2020 Nov 20;10:20243. doi: 10.1038/s41598-020-76791-y (PMC7679461; doi:10.1038/s41598-020-76791-y)
Supplement: Supplementary file 4 — Supplementary Table S2. [file 41598_2020_76791_MOESM4_ESM.docx]

Table S2 Genomic alterations in patients with different smoking status

| Smoking | | | Nonsmoking | | |
| --- | --- | --- | --- | --- | --- |
| Genes | Alteration Number | Mutational Frequency | Genes | Alteration Number | Mutational Frequency |
| TP53 | 44 | 62.86% | EGFR | 72 | 65.45% |
| EGFR | 20 | 28.57% | TP53 | 58 | 52.73% |
| KRAS | 12 | 17.14% | RB1 | 13 | 11.82% |
| CDKN2A | 11 | 15.71% | RBM10 | 11 | 10.00% |
| LRP1B | 9 | 12.86% | SDHA | 11 | 10.00% |
| ALK | 7 | 10.00% | TERT | 10 | 9.09% |
| BCL2L11 | 7 | 10.00% | KRAS | 8 | 7.27% |
| KEAP1 | 7 | 10.00% | CDK4 | 7 | 6.36% |
| KMT2C | 7 | 10.00% | PIK3CA | 7 | 6.36% |
| PIK3CA | 7 | 10.00% | BCL2L11 | 6 | 5.45% |
| STK11 | 7 | 10.00% | LRP1B | 6 | 5.45% |
| FAT3 | 6 | 8.57% | NKX2-1 | 6 | 5.45% |
| NFE2L2 | 6 | 8.57% | ALK | 5 | 4.55% |
| SPTA1 | 6 | 8.57% | BRAF | 5 | 4.55% |
| CCND1 | 5 | 7.14% | EP300 | 5 | 4.55% |
| FAT1 | 5 | 7.14% | GLI1 | 5 | 4.55% |
| FGFR1 | 5 | 7.14% | MET | 5 | 4.55% |
| LRP2 | 5 | 7.14% | APC | 4 | 3.64% |
| SMARCA4 | 5 | 7.14% | ATM | 4 | 3.64% |
| BRCA2 | 4 | 5.71% | CFTR | 4 | 3.64% |
| CCNE1 | 4 | 5.71% | ERBB2 | 4 | 3.64% |
| CDKN2B | 4 | 5.71% | FRS2 | 4 | 3.64% |
| FAM135B | 4 | 5.71% | GNAS | 4 | 3.64% |
| FAT4 | 4 | 5.71% | KMT2D | 4 | 3.64% |
| FGF19 | 4 | 5.71% | MDM2 | 4 | 3.64% |
| FGF3 | 4 | 5.71% | MED12 | 4 | 3.64% |
| FGF4 | 4 | 5.71% | SETD2 | 4 | 3.64% |
| KMT2D | 4 | 5.71% | SPTA1 | 4 | 3.64% |
| MET | 4 | 5.71% | TSC2 | 4 | 3.64% |
| NF1 | 4 | 5.71% | ARFRP1 | 3 | 2.73% |
| POLE | 4 | 5.71% | BCOR | 3 | 2.73% |
| PTEN | 4 | 5.71% | BRIP1 | 3 | 2.73% |
| RBM10 | 4 | 5.71% | CDKN2A | 3 | 2.73% |
| ROS1 | 4 | 5.71% | CTNNB1 | 3 | 2.73% |
| SLIT2 | 4 | 5.71% | FAT4 | 3 | 2.73% |
| SOX2 | 4 | 5.71% | HDAC9 | 3 | 2.73% |
| SPINK1 | 4 | 5.71% | KEAP1 | 3 | 2.73% |
| TERT | 4 | 5.71% | KMT2C | 3 | 2.73% |
| AKT2 | 3 | 4.29% | LRP2 | 3 | 2.73% |
| APC | 3 | 4.29% | MACC1 | 3 | 2.73% |
| ARID1B | 3 | 4.29% | MLH1 | 3 | 2.73% |
| ARID2 | 3 | 4.29% | MTOR | 3 | 2.73% |
| ASXL1 | 3 | 4.29% | MYC | 3 | 2.73% |
| CDK12 | 3 | 4.29% | OBSCN | 3 | 2.73% |
| CTNNB1 | 3 | 4.29% | PALB2 | 3 | 2.73% |
| DNMT3A | 3 | 4.29% | PBRM1 | 3 | 2.73% |
| EPHA3 | 3 | 4.29% | PTEN | 3 | 2.73% |
| ERBB4 | 3 | 4.29% | PTK6 | 3 | 2.73% |
| IKZF1 | 3 | 4.29% | RET | 3 | 2.73% |
| KLHL6 | 3 | 4.29% | RPTOR | 3 | 2.73% |
| MCL1 | 3 | 4.29% | SRMS | 3 | 2.73% |
| NCOR1 | 3 | 4.29% | STK11 | 3 | 2.73% |
| NOTCH2 | 3 | 4.29% | TAF1 | 3 | 2.73% |
| NRG3 | 3 | 4.29% | TRIO | 3 | 2.73% |
| NTRK3 | 3 | 4.29% | APOBEC3B | 2 | 1.82% |
| OBSCN | 3 | 4.29% | AR | 2 | 1.82% |
| RET | 3 | 4.29% | ARID2 | 2 | 1.82% |
| SMAD4 | 3 | 4.29% | BCL6 | 2 | 1.82% |
| SPEN | 3 | 4.29% | BRCA1 | 2 | 1.82% |
| ADGRA2 | 2 | 2.86% | CAMTA1 | 2 | 1.82% |
| AR | 2 | 2.86% | CBFB | 2 | 1.82% |
| ARID1A | 2 | 2.86% | CCND3 | 2 | 1.82% |
| ATM | 2 | 2.86% | CDK12 | 2 | 1.82% |
| AURKA | 2 | 2.86% | CHEK1 | 2 | 1.82% |
| AXL | 2 | 2.86% | CREBBP | 2 | 1.82% |
| BARD1 | 2 | 2.86% | CUL3 | 2 | 1.82% |
| BCL6 | 2 | 2.86% | DNMT3A | 2 | 1.82% |
| BRAF | 2 | 2.86% | ERRFI1 | 2 | 1.82% |
| BRD4 | 2 | 2.86% | FAT3 | 2 | 1.82% |
| CARD11 | 2 | 2.86% | GLI3 | 2 | 1.82% |
| CDK4 | 2 | 2.86% | HMGA2 | 2 | 1.82% |
| CIC | 2 | 2.86% | IL7R | 2 | 1.82% |
| CREBBP | 2 | 2.86% | INHBA | 2 | 1.82% |
| DDR2 | 2 | 2.86% | KEL | 2 | 1.82% |
| EP300 | 2 | 2.86% | KLHL6 | 2 | 1.82% |
| EPHA5 | 2 | 2.86% | LIMK1 | 2 | 1.82% |
| EPHB1 | 2 | 2.86% | MAP3K13 | 2 | 1.82% |
| ERBB2 | 2 | 2.86% | MSH6 | 2 | 1.82% |
| EZH2 | 2 | 2.86% | MUC16 | 2 | 1.82% |
| FANCA | 2 | 2.86% | NF2 | 2 | 1.82% |
| FANCD2 | 2 | 2.86% | NOTCH4 | 2 | 1.82% |
| FRS2 | 2 | 2.86% | PARP4 | 2 | 1.82% |
| GLI3 | 2 | 2.86% | PDGFB | 2 | 1.82% |
| GRM3 | 2 | 2.86% | PDGFRA | 2 | 1.82% |
| HDAC9 | 2 | 2.86% | PIK3C2B | 2 | 1.82% |
| LZTR1 | 2 | 2.86% | POLE | 2 | 1.82% |
| MACC1 | 2 | 2.86% | RAD21 | 2 | 1.82% |
| MAP3K13 | 2 | 2.86% | RAD50 | 2 | 1.82% |
| MDM2 | 2 | 2.86% | ROS1 | 2 | 1.82% |
| MLH1 | 2 | 2.86% | RSPO2 | 2 | 1.82% |
| MYCN | 2 | 2.86% | RUNX1T1 | 2 | 1.82% |
| NKX2-1 | 2 | 2.86% | SETBP1 | 2 | 1.82% |
| NOTCH1 | 2 | 2.86% | SMAD4 | 2 | 1.82% |
| NOTCH3 | 2 | 2.86% | SPINK1 | 2 | 1.82% |
| NOTCH4 | 2 | 2.86% | TET2 | 2 | 1.82% |
| NRAS | 2 | 2.86% | USP6 | 2 | 1.82% |
| PPP2R1A | 2 | 2.86% | VEGFA | 2 | 1.82% |
| PRKCI | 2 | 2.86% | ZNF217 | 2 | 1.82% |
| RANBP2 | 2 | 2.86% | ABL1 | 1 | 0.91% |
| RB1 | 2 | 2.86% | AKT1 | 1 | 0.91% |
| RICTOR | 2 | 2.86% | AKT2 | 1 | 0.91% |
| RIT1 | 2 | 2.86% | AKT3 | 1 | 0.91% |
| RUNX1 | 2 | 2.86% | ARAF | 1 | 0.91% |
| RUNX1T1 | 2 | 2.86% | ARHGEF10 | 1 | 0.91% |
| SDHA | 2 | 2.86% | ARHGEF17 | 1 | 0.91% |
| TAF1 | 2 | 2.86% | ARHGEF25 | 1 | 0.91% |
| TBX3 | 2 | 2.86% | ARHGEF3 | 1 | 0.91% |
| TET1 | 2 | 2.86% | ATRX | 1 | 0.91% |
| TSHR | 2 | 2.86% | AURKA | 1 | 0.91% |
| ZNF217 | 2 | 2.86% | BCR | 1 | 0.91% |
| ZNF703 | 2 | 2.86% | BRCA2 | 1 | 0.91% |
| ABCB1 | 1 | 1.43% | CARD11 | 1 | 0.91% |
| ARHGDIA | 1 | 1.43% | CCND1 | 1 | 0.91% |
| ATRX | 1 | 1.43% | CD1A | 1 | 0.91% |
| AXIN1 | 1 | 1.43% | CDK6 | 1 | 0.91% |
| B2M | 1 | 1.43% | CDKN1A | 1 | 0.91% |
| BCR | 1 | 1.43% | CDKN2B | 1 | 0.91% |
| BLM | 1 | 1.43% | CHD2 | 1 | 0.91% |
| BRCA1 | 1 | 1.43% | CHD4 | 1 | 0.91% |
| BRIP1 | 1 | 1.43% | CIC | 1 | 0.91% |
| CAMTA1 | 1 | 1.43% | CRLF2 | 1 | 0.91% |
| CASP8 | 1 | 1.43% | CUL4A | 1 | 0.91% |
| CBFB | 1 | 1.43% | CYP2D6 | 1 | 0.91% |
| CCND3 | 1 | 1.43% | DDR1 | 1 | 0.91% |
| CD1A | 1 | 1.43% | DICER1 | 1 | 0.91% |
| CD79A | 1 | 1.43% | DNMT3B | 1 | 0.91% |
| CHD2 | 1 | 1.43% | DPYD | 1 | 0.91% |
| COL1A1 | 1 | 1.43% | EPHA6 | 1 | 0.91% |
| CYLD | 1 | 1.43% | EPHA7 | 1 | 0.91% |
| DDR1 | 1 | 1.43% | ERBB3 | 1 | 0.91% |
| DICER1 | 1 | 1.43% | ERBB4 | 1 | 0.91% |
| DIS3 | 1 | 1.43% | ETV1 | 1 | 0.91% |
| DOT1L | 1 | 1.43% | ETV5 | 1 | 0.91% |
| EMSY | 1 | 1.43% | ETV6 | 1 | 0.91% |
| ERBB3 | 1 | 1.43% | EWSR1 | 1 | 0.91% |
| ERCC1 | 1 | 1.43% | FAM135B | 1 | 0.91% |
| ERG | 1 | 1.43% | FAM46C | 1 | 0.91% |
| ERRFI1 | 1 | 1.43% | FANCA | 1 | 0.91% |
| ETV1 | 1 | 1.43% | FANCC | 1 | 0.91% |
| ETV6 | 1 | 1.43% | FANCD2 | 1 | 0.91% |
| EZR | 1 | 1.43% | FANCF | 1 | 0.91% |
| FANCM | 1 | 1.43% | FANCG | 1 | 0.91% |
| FBXW7 | 1 | 1.43% | FANCL | 1 | 0.91% |
| FEV | 1 | 1.43% | FGF1 | 1 | 0.91% |
| FGF10 | 1 | 1.43% | FGF10 | 1 | 0.91% |
| FGF14 | 1 | 1.43% | FGF19 | 1 | 0.91% |
| FLCN | 1 | 1.43% | FGF3 | 1 | 0.91% |
| FLT3 | 1 | 1.43% | FGF4 | 1 | 0.91% |
| FLT4 | 1 | 1.43% | FGF5 | 1 | 0.91% |
| FUS | 1 | 1.43% | FGFR2 | 1 | 0.91% |
| GATA6 | 1 | 1.43% | FGFR3 | 1 | 0.91% |
| GLI1 | 1 | 1.43% | FH | 1 | 0.91% |
| GNAS | 1 | 1.43% | FLI1 | 1 | 0.91% |
| GRIN2A | 1 | 1.43% | FLT4 | 1 | 0.91% |
| HCK | 1 | 1.43% | FUS | 1 | 0.91% |
| HMGA2 | 1 | 1.43% | GATA6 | 1 | 0.91% |
| IL7R | 1 | 1.43% | GLI2 | 1 | 0.91% |
| INPP4B | 1 | 1.43% | GRIN2A | 1 | 0.91% |
| IRS2 | 1 | 1.43% | GRM3 | 1 | 0.91% |
| KAT6A | 1 | 1.43% | HGF | 1 | 0.91% |
| KDM5C | 1 | 1.43% | HSD3B1 | 1 | 0.91% |
| KEL | 1 | 1.43% | INPP4B | 1 | 0.91% |
| KIT | 1 | 1.43% | ITK | 1 | 0.91% |
| MAGI2 | 1 | 1.43% | KDM5A | 1 | 0.91% |
| MAP2K1 | 1 | 1.43% | KDM5C | 1 | 0.91% |
| MAP2K4 | 1 | 1.43% | KDR | 1 | 0.91% |
| MED12 | 1 | 1.43% | LRP1 | 1 | 0.91% |
| MEN1 | 1 | 1.43% | LZTR1 | 1 | 0.91% |
| MST1R | 1 | 1.43% | MAGI2 | 1 | 0.91% |
| MUC16 | 1 | 1.43% | MAP3K1 | 1 | 0.91% |
| MYC | 1 | 1.43% | MSH2 | 1 | 0.91% |
| NCOA2 | 1 | 1.43% | MST1R | 1 | 0.91% |
| NSD1 | 1 | 1.43% | MTAP | 1 | 0.91% |
| NTRK1 | 1 | 1.43% | MUTYH | 1 | 0.91% |
| PALB2 | 1 | 1.43% | MYCL | 1 | 0.91% |
| PBRM1 | 1 | 1.43% | MYOD1 | 1 | 0.91% |
| PDGFRA | 1 | 1.43% | NCOA2 | 1 | 0.91% |
| PIK3C3 | 1 | 1.43% | NCOR1 | 1 | 0.91% |
| PIK3CB | 1 | 1.43% | NET1 | 1 | 0.91% |
| PIK3R2 | 1 | 1.43% | NF1 | 1 | 0.91% |
| PLCG2 | 1 | 1.43% | NFE2L2 | 1 | 0.91% |
| PMS2 | 1 | 1.43% | NOTCH3 | 1 | 0.91% |
| POLB | 1 | 1.43% | NRG3 | 1 | 0.91% |
| PREX2 | 1 | 1.43% | NTRK1 | 1 | 0.91% |
| PRSS8 | 1 | 1.43% | NTRK3 | 1 | 0.91% |
| PTCH1 | 1 | 1.43% | PARP1 | 1 | 0.91% |
| PTPN11 | 1 | 1.43% | PAX5 | 1 | 0.91% |
| RAC1 | 1 | 1.43% | PBX1 | 1 | 0.91% |
| RAD51B | 1 | 1.43% | PDGFRB | 1 | 0.91% |
| REL | 1 | 1.43% | PDK1 | 1 | 0.91% |
| RELA | 1 | 1.43% | PIK3C2G | 1 | 0.91% |
| SETBP1 | 1 | 1.43% | PIK3CB | 1 | 0.91% |
| SETD2 | 1 | 1.43% | PIK3CD | 1 | 0.91% |
| SF3B1 | 1 | 1.43% | PIK3CG | 1 | 0.91% |
| SMAD3 | 1 | 1.43% | PMS2 | 1 | 0.91% |
| SMARCB1 | 1 | 1.43% | PREX2 | 1 | 0.91% |
| SMO | 1 | 1.43% | PRSS8 | 1 | 0.91% |
| SRSF2 | 1 | 1.43% | PTCH1 | 1 | 0.91% |
| SS18 | 1 | 1.43% | QKI | 1 | 0.91% |
| TCF7L2 | 1 | 1.43% | RAC1 | 1 | 0.91% |
| TET2 | 1 | 1.43% | RAD52 | 1 | 0.91% |
| TNFAIP3 | 1 | 1.43% | RAD54B | 1 | 0.91% |
| TNK2 | 1 | 1.43% | RARA | 1 | 0.91% |
| TP63 | 1 | 1.43% | REL | 1 | 0.91% |
| TRAF7 | 1 | 1.43% | RELA | 1 | 0.91% |
| TRIO | 1 | 1.43% | REV3L | 1 | 0.91% |
| TSC1 | 1 | 1.43% | RHOA | 1 | 0.91% |
| USP6 | 1 | 1.43% | ROCK1 | 1 | 0.91% |
| VEGFA | 1 | 1.43% | RUNX1 | 1 | 0.91% |
| VGLL3 | 1 | 1.43% | SDC4 | 1 | 0.91% |
| WT1 | 1 | 1.43% | SF3B1 | 1 | 0.91% |
| XPO1 | 1 | 1.43% | SKP2 | 1 | 0.91% |
| XRCC3 | 1 | 1.43% | SLIT2 | 1 | 0.91% |
| YES1 | 1 | 1.43% | SMARCA4 | 1 | 0.91% |
| ZNF750 | 1 | 1.43% | SOX2 | 1 | 0.91% |
| (空白) |  | 0.00% | SPEN | 1 | 0.91% |
|  |  |  | SRC | 1 | 0.91% |
|  |  |  | TBX3 | 1 | 0.91% |
|  |  |  | TFE3 | 1 | 0.91% |
|  |  |  | TFEB | 1 | 0.91% |
|  |  |  | TGFBR2 | 1 | 0.91% |
|  |  |  | TIE1 | 1 | 0.91% |
|  |  |  | TMPRSS2 | 1 | 0.91% |
|  |  |  | TNFRSF14 | 1 | 0.91% |
|  |  |  | TNFSF11 | 1 | 0.91% |
|  |  |  | TOP1 | 1 | 0.91% |
|  |  |  | TP63 | 1 | 0.91% |
|  |  |  | TSC1 | 1 | 0.91% |
|  |  |  | TSHR | 1 | 0.91% |
|  |  |  | TSPAN31 | 1 | 0.91% |
|  |  |  | TYRO3 | 1 | 0.91% |
|  |  |  | U2AF1 | 1 | 0.91% |
|  |  |  | WEE1 | 1 | 0.91% |
|  |  |  | WRN | 1 | 0.91% |
|  |  |  | WT1 | 1 | 0.91% |
|  |  |  | XIAP | 1 | 0.91% |
